# Development of Novel *In Vivo* Chemical Probes to Address CNS Protein Kinase Involvement in Synaptic Dysfunction

**DOI:** 10.1371/journal.pone.0066226

**Published:** 2013-06-26

**Authors:** D. Martin Watterson, Valerie L. Grum-Tokars, Saktimayee M. Roy, James P. Schavocky, Brinda Desai Bradaric, Adam D. Bachstetter, Bin Xing, Edgardo Dimayuga, Faisal Saeed, Hong Zhang, Agnieszka Staniszewski, Jeffrey C. Pelletier, George Minasov, Wayne F. Anderson, Ottavio Arancio, Linda J. Van Eldik

**Affiliations:** 1 Department of Molecular Pharmacology and Biological Chemistry, Northwestern University, Chicago, Illinois, United States of America; 2 Sanders-Brown Center on Aging, University of Kentucky, Lexington, Kentucky, United States of America; 3 Department of Pathology and Cell Biology, Columbia University, New York, New York, United States of America; Stanford University, United States of America

## Abstract

Serine-threonine protein kinases are critical to CNS function, yet there is a dearth of highly selective, CNS-active kinase inhibitors for *in vivo* investigations. Further, prevailing assumptions raise concerns about whether single kinase inhibitors can show *in vivo* efficacy for CNS pathologies, and debates over viable approaches to the development of safe and efficacious kinase inhibitors are unsettled. It is critical, therefore, that these scientific challenges be addressed in order to test hypotheses about protein kinases in neuropathology progression and the potential for *in vivo* modulation of their catalytic activity. Identification of molecular targets whose *in vivo* modulation can attenuate synaptic dysfunction would provide a foundation for future disease-modifying therapeutic development as well as insight into cellular mechanisms. Clinical and preclinical studies suggest a critical link between synaptic dysfunction in neurodegenerative disorders and the activation of p38αMAPK mediated signaling cascades. Activation in both neurons and glia also offers the unusual potential to generate enhanced responses through targeting a single kinase in two distinct cell types involved in pathology progression. However, target validation has been limited by lack of highly selective inhibitors amenable to *in vivo* use in the CNS. Therefore, we employed high-resolution co-crystallography and pharmacoinformatics to design and develop a novel synthetic, active site targeted, CNS-active, p38αMAPK inhibitor (MW108). Selectivity was demonstrated by large-scale kinome screens, functional GPCR agonist and antagonist analyses of off-target potential, and evaluation of cellular target engagement. *In vitro* and *in vivo* assays demonstrated that MW108 ameliorates beta-amyloid induced synaptic and cognitive dysfunction. A serendipitous discovery during co-crystallographic analyses revised prevailing models about active site targeting of inhibitors, providing insights that will facilitate future kinase inhibitor design. Overall, our studies deliver highly selective *in vivo* probes appropriate for CNS investigations and demonstrate that modulation of p38αMAPK activity can attenuate synaptic dysfunction.

## Introduction

An evolving view of progressive neurodegenerative disorders such as Alzheimer’s disease (AD), frontotemporal dementia, and Parkinson’s disease is their potential to be approached as diseases of progressive synaptic dysfunction [1]–[3]. Considerable clinical and preclinical studies suggest a critical link between the synaptic dysfunction in neurodegenerative disorders and activation of p38MAPK signaling cascades in both neurons and glia [4], [5]. Mammals have four different genes that encode distinct p38 MAPK isoforms (p38α, p38β, p38γ, p38δ) with high sequence similarity (e.g, p38α has approximately 75% sequence identity to p38β). The four p38MAPK isoforms are widely expressed and selectively initiate downstream responses dependent on the cell type and the activating stimulus. In neurodegenerative disease models, specific p38MAPK signaling cascades involving substrates such as MAPKAP-K2 (MK2) and tau have been implicated in controlling neuroinflammation, neuronal plasticity, and synaptic/dendritic pathology [4], [5]. However, validation of p38αMAPK as a potential CNS drug discovery target has been limited by the lack of highly selective p38αMAPK inhibitors amenable to *in vivo* use in the CNS.

A variety of small molecules targeting p38MAPKs allowed pursuit of *in vitro* studies and *in vivo* investigations of pathology progression in some peripheral tissues, but the pharmacokinetics (what the body does to the molecule) precluded their full use for *in vivo* studies of CNS tissue pharmacodynamics (what the molecule does to the body). Generally, the molecular properties of the inhibitors did not allow adequate CNS exposure, thereby limiting their potential use in CNS investigations [6], [7]. In other studies, interpretation of the pharmacodynamics was hindered by the multi-kinase nature of the inhibitors [8], [9]. For example, there are literally hundreds of reports using commercially available inhibitors such as SB239063 and SB203580, but like many p38αMAPK inhibitors these inhibit casein kinase 1δ (CK1δ), p38βMAPK and other kinases [8]–[10]. Although no selective CNS penetrant p38αMAPK has yet progressed to human studies, the ambiguity of results from non-CNS clinical investigations and preclinical studies raises concerns. For example, an appropriate pharmacodynamic effect in arthritis investigations was observed (decrease of proinflammatory cytokine levels back towards normal), but efficacy in disease alteration was not evident [11]. Although the reasons for the desired but transient pharmacodynamics with efficacy failure in non-CNS disorders are not known, diverse mechanisms (e.g., tachyphylaxis) are logical to anticipate in extended studies where repeat administrations of a pleotropic drug are involved. Clearly, there is a critical need for selective p38 MAPK inhibitors that are also appropriate for the study of CNS protein kinases if we are to better understand how regulatory nodes in complex signaling networks are linked to potential mechanisms of pathology progression and alteration.

Two current approaches to generate selective small molecule kinase inhibitors are the allosteric approach, which involves induction or prevention of enzyme conformational changes via targeting sites outside the catalytic region, and the active site approach [12]–[14]. The allosteric approach is an area of active investigation that has yet to be generally validated or reduced to standard practice. The active site approach is that used successfully to deliver new classes of approved therapeutics and generate the vast majority of extant molecular probe reagents for *in vivo* studies in diverse phylogenetic species. However, the kinase inhibitors generated by the active site approach, although highly restrictive in nature, are still generally multi-kinase inhibitors. The multi-kinase nature and failures with many current kinase inhibitors have led to a prevailing assumption that efficacy might not be achievable by targeting a single kinase [15].

This hypothesis has been difficult to test because of the lack of appropriate inhibitors.

A recently emerging variant of the active site approach focuses on induction of localized conformational changes by inhibitors in order to achieve selectivity [16]–[30]. In the case of p38αMAPK inhibitors, the approach posits that the common theme of exploiting hydrogen (H)-bond interactions with the Met109 amide bond in the phylogenetically conserved kinase hinge region can be enhanced by designing inhibitors that can also engage the contiguous Gly110, thereby yielding highly selective inhibitors that can induce a localized conformational change, termed a glycine flip. The approach is based on the finding that only a few kinases have a Gly contiguous to the point of H-bond interaction. However, the paucity of p38αMAPK inhibitors that lack problematic cross-over to other serine-threonine kinases, such as CK1δ, and their evaluation by high-resolution crystallography preclude robust evaluation of such hypotheses as a design strategy.

The p38αMAPK is of neuropharmacology interest as it is associated with intracellular signal transduction in both glia and neurons under stresses that are linked to progressive synaptic dysfunction. Therefore, a highly selective p38αMAPK inhibitor might be targeting a physiological axis, offering the potential of pharmacological synergy where dose dependent inhibition of the same target in each cell type might generate a more robust response than in other pathophysiology paradigms. As a starting point for such *in vivo* investigations, we developed a set of highly selective inhibitors appropriate for CNS investigations and used the compounds to implicate p38αMAPK as a potential target for intervention and attenuation of synaptic dysfunction. The probes include an isoform-selective p38αMAPK inhibitor, MW01-11-108 ( = MW108) and a p38MAPK subfamily inhibitor MW01-10-181SRM ( = MW181). The high level of selectivity is indicated by large-scale kinome activity screens, functional GPCR cell-based evaluation for agonist and antagonist activity, and cellular target engagement analyses. *In vitro* and *in vivo* assays demonstrated the ability of MW108 and MW181 to ameliorate beta-amyloid induced synaptic and cognitive dysfunction.

High-resolution crystallographic analysis of human p38αMAPK in complex with the new selective inhibitors confirmed the active site targeting and, interestingly, demonstrated the lack of a detectable glycine flip in the structures. Therefore, control experiments on the structure of human p38αMAPK with active site bound SB239063, a mixed-kinase inhibitor, were performed and demonstrated the glycine flip conformation. The finding that the less selective p38 inhibitor SB239063 can induce the glycine flip, whereas MW108 and MW181 do not, indicates that the ability to induce such a localized conformational change is not a prerequisite for active site targeted probes with greater specificity. Overall, the probes described here represent valuable chemical biology tools for future *in vivo* investigations, and provide structural insights that may facilitate future active site targeted inhibitor design.

.

## Materials and Methods

### Synthesis of Novel, Selective p38αMAPK Inhibitors

**Figure 1:**
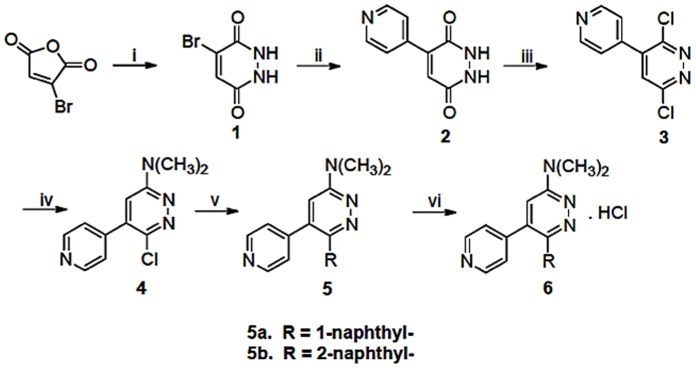
Discovery synthetic scheme for title compounds. Reagents and conditions: i) Hydrazine sulphate, H_2_O, 100°C; ii) Pyridin-4-ylboronic acid; 1,2-dimethoxyethane (DME)/water, Na_2_CO_3_, Pd(PPh_3_)_4_, 110°C; iii) phosphorus oxychloride/acetonitrile, 90°C; iv) Ethanol, amine, reflux; v) 1- or 2- Naphthylboronic acid, DME/water, Na_2_CO_3_, Pd(PPh_3_)_4_, 110°C; vi) concentrated HCl, anhydrous isopropanol, 80°C.

The synthetic scheme and experimental details for the two novel title compounds, **MW01-10-181SRM** (**MW181**) and **MW01-11-108SRM** (**MW108**), are shown in **Figure 1** in sufficient detail for reproduction by one skilled in the art. The design strategy and synthesis of additional compounds used in the investigation are presented in **Figure S1 and** **Scheme S1**.

Chemicals were purchased from Aldrich (Milwaukee, WI) or VWR International (Batavia, IL). Solvents were used as received unless stated otherwise. Water was obtained using a Milli-Q Biocel A10 purification system from Millipore Corporation (Bedford, MA). High pressure glassware was from Chemglass, (Vineland, NJ). All syntheses were monitored by HPLC (Dionex System, Sunnyvale, CA, with UVD170U ultraviolet detector and P680 pump) on a Phenomenex (Torrance, CA) Luna C18 column (250×2.0 mm; 5 µm; equipped with guard column) at a flow rate of 0.2 mL/min and using a mobile phase composed of 0.1% (v/v) formic acid (Fluka) in water as reagent A and 80% acetonitrile, 0.08% formic acid/water as reagent B. Synthetic intermediates were screened by electrospray mass spectroscopy (ESI) HPLC (Micromass Quattro II Triple Quadrupole HPLC/MS/MS mass spectrometer). Final compounds were analyzed by HPLC, high-resolution mass spectra (HRMS; VG70-250SE mass spectrometer) and ^1^H NMR (Bruker Avance-III 500 MHz spectrometer; ambient temperature). Melting point data for compounds were acquired on a Büchi Melting Point B-540 (Flawil, Switzerland). Hydrochloride hydrates (salts) of final compounds were characterized by elemental analysis (Intertek QTI; Whitehouse, NJ).

#### 4-bromo-1,2-dihydropyridazine-3,6-dione (1)

Hydrazine sulfate (2.25 g, 17.2 mmol) was dissolved in boiling water (20 mL) with stirring. To this solution, bromomaleic anhydride (2.6ml, 28.2 mmol) was added drop wise via addition funnel, the mixture heated (100^o^C) under reflux for 19 h, then cooled to ambient temperature. The resulting white precipitate was filtered on a medium frit sintered glass funnel, washed with acetone (3×5 ml), and air dried *in vacuo* to give the desired product 4-bromo-1,2-dihydropyridazine-3,6-dione (2.85 g) as a white powder in 87% yield (gravimetric) with a melting point of 262°C.

#### 4-(pyridin-4-yl)-1,2-dihydropyridazine-3,6-dione (2)

Essentially as previously described [31], compound **1** (2 g, 10.4 mmol, 1 eq) and pyridin-4-yl boronic acid (14.3 mmol, 1.76 g, 1.37 eq) were suspended in dimethoxyethane and water (10∶1 v/v) in a heavy wall pressure vessel and purged with argon for 15 min. Tetrakis (triphenylphosphine) palladium (0.1 eq) and sodium carbonate (3 eq) were added, the vessel immediately capped, the reaction mixture heated (110°C ) for 18 h, then cooled to ambient temperature and subjected to filtration on a medium frit sintered glass funnel containing Celite® 545. The filtrate was concentrated *in vacuo* and the concentrate triturated with hexane. The yellow product **2** (2.2 g) exhibited a mass (ESI) of *m/z* (MeOH) = 190.06 (MH^+^), and was taken to the next step without further purification.

#### 3,6-dichloro-4-(pyridin-4-yl)pyridazine (3)

Essentially as described [32], compound **2** (2.2 g, 11.6 mmol) was suspended in 6.25 ml phosphorus oxychloride in a condenser-fitted round bottom flask, heated (90°C) for 24 h, cooled to ambient temperature and volatiles removed *in vacuo.* The dark residue was poured onto crushed ice, stirred (2 h), and the mixture neutralized with saturated sodium carbonate solution. The fine precipitate was subjected to replicate extraction with dichloromethane in a separatory funnel, the combined organic phases subjected to drying over anhydrous sodium sulfate, concentrated *in vacuo*, and subjected to column chromatography on silica gel (200-400 mesh) using a ethyl acetate:hexane (3∶2 v/v) eluent. The desired product **3** exhibited 97% purity by HPLC and a mass (ESI) of *m/z* (MeOH) = 225.99 (MH^+^). The overall yield (gravimetric) from product **1** to **3** was approximately 36%.

#### 6-chloro-N,N-dimethyl-5-(pyridin-4-yl)pyridazin-3-amine (4)

Following published protocol [32], compound **3** (11.06 mmol, 1 eq) in ethanol (60 ml) was placed in a heavy wall pressure vessel and 40% dimethylamine (6 eq) added, the vessel capped and heated (110°C) for 16 h, the mixture allowed to cool to ambient temperature then transferred to a round bottom flask for concentration *in vacuo.* The residue was treated with 10 ml of water, the aqueous phase subjected to replicate extraction with dichloromethane in a separatory funnel, and the combined organic layers dried (anhydrous sodium sulfate) and evaporated under reduced pressure to yield a yellow adherent solid. The reaction mixture was purified by silica gel (200–400 mesh) column chromatography and the desired product eluted with ethyl acetate:hexane (1∶1 v/v). Product **4** was obtained as a yellow solid in 89% (gravimetric) yield, with an apparent HPLC purity of 98% and a mass (ESI) m/z (MeOH) = 235.10 (MH^+^).

#### N,N-dimethyl-6-(naphthalen-1-yl)-5-(pyridin-4-yl)pyridazin-3-amine (5a = MW181)

Compound **4** (1.95 g, 8.5 mmol, 1 eq) and 1-naphthylboronic acid (1.96 g, 11.3 mmol, 1.37 eq) were suspended in dimethoxyethane (DME) and water (10∶1, v/v) in a heavy wall pressure vessel and purged with argon for 15 min. Tetrakis (triphenylphosphine) palladium (0.860 g, 9 mol%) and Na_2_CO_3_ (2.7 g, 25.7 mmol, 3.1 eq) were added, the vessel purged with argon and immediately capped with a Teflon bushing, heated (110°C) for 17 hr, and cooled to ambient temperature. The reaction mixture was subjected to filtration on a medium frit sintered glass funnel containing Celite® 545. The resultant filtrate was concentrated *in vacuo,* the residue dissolved in CH_2_Cl_2_, and subjected to water extraction (3×30 ml) in a separatory funnel. The organic layer was dried over anhydrous sodium sulfate and concentrated by rotary evaporation under reduced pressure. The crude mixture was subjected to silica gel (200–400 mesh) column chromatography with product elution using ethyl acetate: hexane (2∶3 v/v). The product was crystallized from hexane and ethyl acetate. Product **5a** **(MW181)** was obtained as light yellow crystals in 56% yield (gravimetric). HPLC purity, 98%; ESI m/z (MeOH), 327.10 (MH+); MP = 189.5–190°C (uncorrected); HRMS, 326.1526 (calculated for C_21_H_18_N_4_ = 326.1531). ^1^H-NMR (CDCl3): 8.34 (d, J = 5.8Hz, 2H); 7.83(d, J = 8.2Hz, 2H); 7.68 (d, J = 8.4Hz, 1H); 7.42–7.26(m, 4H); 6.99 (dd, J = 1.5, 4.6, 2H); 6.84 (s, 1H); 3.33(s, 6H).

#### N, N-dimethyl-6-(naphthalen-2-yl)-5-(pyridin-4-yl)pyridazin-3-amine (5b = MW108)

Product **5b** was produced using the protocol as above for **5a** but using 2-naphthylboronic acid (595 mg, 2.9 mmol, 1.37 eq) suspended in DME:water mixture (10∶1, v/v) The final product, **5b (MW-108)** was obtained as a whitish powder in 52% yield (gravimetric). HPLC purity, 96%; ESI m/z (MeOH), 327.10 (MH+); MP = 181.5–182°C (uncorrected); HRMS, 326.1538 (calculated for C_21_H_18_N_4_ = 326.1531). ^1^H-NMR (CDCl_3_): ^1^H-NMR (CDCl_3_): 8.55 (d, J = 5.65 Hz, 2H); 7.93(s, 1H); 7.80–7.68 (m, 3H); 7.49–7.34(m, 3H); 7.19 (d, J = 5.95Hz, 2H); 6.76 (s, 1H); 3.30(s, 6H).

#### Hydrochloride hydrate (6) of products 5a and 5b

This was done as described [32]. Briefly, approximately 1 mmol of the respective compound was suspended in 8.2 ml of anhydrous isopropanol (99.5%, Aldrich), heated to 85°C with stirring until dissolved, then 3.1 equiv (234µl) of concentrated ultrapure HCl (12N, JT Baker Ultrex® II, Product 6900-05) was added to the clear solution, resulting in immediate generation of a suspension of solids. The suspension was stirred for 10 min (80°C), allowed to cool to ambient temperature, then the vessel placed on ice for ∼2.5 h, then stored (∼16 hr) at 4°C. The resulting yellow precipitate was filtered on a medium frit sintered glass funnel under vacuum, washed (3x) with cold anhydrous isopropanol followed by three washes with cold anhydrous ether, and dried under vacuum. The precipitate was stored *in vacuo* in a glass desiccator containing silica gel until a constant weight was attained. The final products were obtained in approximately 82% yield (gravimetric) compared to the starting material. Hydrochloride hydrate formation was confirmed by elemental analysis. The goal was to obtain a mole ratio of HCl:compound that is >1. Under these conditions for the studies described here, elemental analysis indicated a ratio of ∼2. EA calculated for C_21_H_24_Cl_2_N_4_O_2_ is: C, 57.94; H, 5.56; N, 12.87; Cl, 16.29; O, 7.35. Experimentally found for MW-181: C, 58.16; H, 5.60; N, 12.51; Cl, 16.52; O, 7.37. Experimentally found for MW-108: C, 56.09; H, 5.65; N, 12.38; Cl, 15.91; O, 10.16. Of special note is the color of the final product, which is light yellow. A darker color can be a potential indicator of non-organic impurities, such as the presence of residual acid.

### Chemical Characterization of Title Compounds MW108 and MW181

Aqueous solubility (logS) and chemical stability were determined as previously described [32]. Briefly, solutions for aqueous solubility (log S) determination were made by gravimetric methods using a Sartorius AG (Germany) BP 211 D analytical balance. Dry borosilicate capillary tubes (Büchi, Switzerland) were weighed and various amounts of compound (ranging from 1.2–1.5 mg) or the hydrochloride hydrate form (ranging from 10–20 mg) were added to the tared tubes, then water added to create solutions ranging in concentrations from 1–2 g/mL. Tubes containing the samples were mixed to ensure wetting and incubated at 37°C for 24 h. Aliquots were collected from each tube, centrifuged at 12,000 g-min, and analyzed by HPLC. Concentrations were calculated using area under the peak (260 nm) at the appropriate retention time relative to a standard curve obtained from serial dilutions of each purified compound. As an independent and extended control evaluation of aqueous solubility, MW181 solubility was determined by CEREP, Inc. in simulated gastric fluid by the shake-flask technique. Chemical stability was determined at a final concentration of 0.0015 M at neutral (Milli-Q water), 0.1 N NaOH (pH 13–14), 0.1 N HCl (pH 1–2) conditions with samples taken for analysis at 3, 6, 24 h. As an independent and extended control evaluation of chemical stability, MW181 solubility was determined by CEREP, Inc in simulated gastric fluid at 37°C. The acid dissociation constant (pKa) was determined by pION Inc. (Billerica, MA) using both potentiometric and UV methods. The co-solvent was methanol, experimental pH range was 1.7–10.0, temperature was 22±2°C, ionic strength was 0.15 M KCl. Five titrations were used to calculate the pKa using the Yasuda-Shedlovsky technique [33]. Quality control and calibrants used were phosphate, 0.5 N KOH, 0.5 N HCl, and the FDA approved drug flubiprofen (MW 244.2, PSA 37.3, cLogP 3.7). The screen for ratio of peak concentration of orally administered MW108 or MW181 in blood and brain was done as described previously [31], [32] using an oral gavage dose of 10 mg/kg body weight in sterile saline. Processed samples from tissue extracts were acidified with HPLC limit solvent and subjected to solid phase extraction with elution done with 10% (v/v) acetonitrile into HPLC analysis solvent A. The clinical CNS drug minaprine in its hydrochloride hydrate formulation, in the same 3-amino-6-arylpyridazine scaffold class as MW108 and MW181, was used as control and internal standard.

### Protein Kinase Inhibitor Activity

Concentration-dependent inhibition of protein kinase activity was done essentially as previously described [31]. Assays had calculated Z-factors ≥0.5 and %CV <20%. Briefly, enzyme activity assays were done with p38MAPK/SAPK2 and CK1γ enzymes (Millipore; Billerica, MA) and either the substrate myelin basic protein (MBP, at 0.33 mg/ml for p38MAPKs) or the substrate KRRRALS(p)VASLPGL (200 µM for CK1γ) in either 25 mM Tris pH 7.5, 0.02 mM EGTA or in 50 mM HEPES, pH 7.5, 5 mM MgCl_2_, 150 mM KCl, 15 mM NaCl, 1 mM DTT. Reactions contained 100 µM ATP with γ-[^32^P]ATP (specific activity >300 cpm/pmol). Reactions were initiated by addition of ATP substrate mix or the active enzyme. Reactions were stopped by transfer of aliquots to P81 paper (Whatman, Clifton, NJ), and the paper washed sequentially with 75 mM phosphoric acid and 95% ethanol. Quantification was done by scintillation counting. Inhibitors were made as 10X stock solutions in DMSO or water, and controls contained the same final concentration of solvent added. Data are expressed as percent of the maximal enzyme activity, where enzyme activity in the absence of compound is taken as 100%. IC_50_ values are taken from the midpoint of a 10–11 point curve of replicates that corresponds to 50% inhibition, and were calculated using nonlinear regression curve fit analysis in GraphPad Prism statistical software. The apparent *Km* for ATP was determined by measuring kinase activity at six ATP concentrations (10, 45, 90, 200, 500, 1000 µM) in the absence of inhibitor. To estimate *Ki* values, these ATP concentrations ranging from ten fold above and below the apparent *Km* for ATP (approximately 100 µM) were used with increasing concentrations of inhibitor to generate EC_50_ values taken from the midpoint of the sigmoidal curves. The EC_50_ values were re-plotted against the ATP concentration and the data fitted to a linear regression to obtain the y-axis intercept, which is taken as the *Ki*.

### Hierarchal Large-scale Kinome Analysis

Large-scale kinome screens were done using a hierarchal screening protocol. First, an initial profiling was done with each inhibitor tested at a fixed concentration (20,000 nM) in the commercially available Millipore® Profiler test systems (www.millipore.com) that included >290 mammalian kinases representative of all major kinome branches as well as isoforms of individual families. The list of protein and lipid kinases and their NCBI Entrez accession numbers are provided in **Tables S1** and **S2**. Second, preliminary hits from the profiler screen (<40% kinase activity remaining) were validated as true or false positive hits by a follow-up, concentration-dependent test of the inhibitor to obtain an IC_50_ value for the inhibitor and a given kinase. Third, kinetic analyses were done as described above to determine a *Ki* value on confirmed positives with IC_50_ values <1,000 nM. The full list of mammalian kinases, their NCBI database accession identifier, the profiler screen results (% kinase activity remaining), and the IC_50_ from the concentration-dependent activity assays for hits are provided for MW108 and MW181 in **Tables S1** and **S2**.

### Adverse Pharmacology and Off-target Functional Screens

Novel compounds were screened *in vivo* using a standard dose escalation screen in mice coupled with clinical observation and testing based on the SHIRPA mouse phenotype assay paradigm [34]. To obtain insight into the potential for off-target activity with the largest known family of small molecule drug targets, G-protein coupled receptors (GPCRs), a cell-based functional screen of the final title compounds was done. The Millipore® GPCR Profiler screen employs the ChemiScreen GPCR stable cell line technology used for real-time calcium flux FLIPR assays on a panel of 158 GPCRs to detect both antagonists and agonists. The NCBI Entrez identifier for each GPCR is provided at the vendor site (www.millipore.com). Follow-up validation of any initial hits as being true positives or negatives was done as described above for protein kinases by testing the concentration dependence of any cellular effects. No validated hits were detected.

### Crystallographic Analysis of Human p38αMAPK in Complex with Inhibitors

Human p38αMAPK with an amino terminal His_6_-tag was produced by expression cloning in BL21(DE3)pLysS *E. coli.* After recombinant DNA mediated insertion of the kinase cDNA (NCBI Reference Sequence: NM_139012) region encoding amino acids 2–360 into the pMCSG7 expression vector at the SspI site, transformed cells were selected with ampicillin (100 µg/ml) and chloramphenicol (15 µg/ml), grown at 37°C until the A_600_ = 0.5, then continued at 25°C for 14 hr after addition of isopropyl-β-D-1-thiogalactopyranoside to a final concentration of 1 mM. Cell pellets were collected by centrifugation (5100 *g*; 20 min), immediately frozen at −20°C, rapidly thawed, and subjected to sonication in buffer A (10 mM Tris pH 8.3, 0.5 M NaCl, 5 mM β-mercaptoethanol) containing 10% (v/v) glycerol, 1µM pepstatin A, 10 µM leupeptin, 1µM TLCK. All subsequent purification steps were done at 4°C. The cell lysate was clarified by centrifugation (39,000 *g*; 50 min) and the resultant supernatant loaded onto a 5 ml HisTrap™ column (GE Healthcare, Cat. No. 17-5248-01) equilibrated with buffer A. The column was washed with 5 column volumes of buffer A containing 25 mM imidazole, and step-elution of adsorbed protein done with buffer A containing 0.5 M imidazole. The eluted protein was desalted by passage through a HiPrep 26/10 column (GE Healthcare, Cat. No. 17-5087-01) equilibrated with buffer B (10 mM Tris pH 8.3 and 5 mM β-mercaptoethanol) and subjected immediately to chromatography on a 5 ml Q Sepharose™ High Performance column (GE Healthcare, Cat. No. 17-1154-01) equilibrated with buffer B. Column elution was done using a linear 80 mL gradient from 0–0.5 mM NaCl in buffer B. Concentration of the protein solution to 20 mg/ml in buffer B containing 150 mM NaCl was done using a 10,000 MW cutoff concentrator (Vivascience/Sartorius Cat. No. Vs2001, Aubagne, France). Protein concentration was determined at A = 280 nm using a NanoDrop system (Wilmington, Delaware, USA), and an extinction coefficient of 1.13 (mg/mL)^-1^cm^-1^. The protein solution was monodisperse based on dynamic light scattering (Zetasizer Nanoseries Model Zen1600, Malvern Instruments Ltd; Malvern, Worcestershire, UK) done at 25°C and homogenous by SDS–PAGE. Protein was stored at −80°C as flash frozen aliquots. Properties of frozen and once thawed aliquots were indistinguishable from freshly prepared protein solutions.

Protein samples for crystallization were prepared just before use by adding an equal volume of 4 mM 4-(3-(4-fluorophenyl)-1H-pyrazol-4-yl)pyridine (4-FPP; ASINEX-USA, Cat. No. LEG 22383617) in 8% (v/v) DMSO (Sigma, Cat. No. D-2650) to the previously described p38αMAPK enzyme preparation (20 mg/ml in buffer B containing 150 mM NaCl), and incubated for approximately 45 min (4°C). Screening for crystallization conditions was done with a commercially available kit (Classics II screen; catalog No. 136165; Qiagen, Valencia, California, USA) and the sitting drop method as previously described [35]. Visible crystals were evident in less than 48 hrs at 22°C under conditions of 0.1 M ammonium acetate, 0.1 M bis-tris pH 5.5, 17%(w/v) PEG 10,000.

Formation of complexes with inhibitors MW181, MW108, and SB239063 for crystallographic structure determination was done using the crystal soak approach. Briefly, protein crystals were transferred to individual wells of a Pyrex® clear glass 9-well spot plate (Cat. No. 7220–85; Corning MA) containing 0.1 M ammonium acetate, 0.1 M bis-tris pH 5.5, 17%(w/v) PEG 10,000, and inhibitor was added at a final concentration of 5 mM from a stock solution of 50 mM in either saline (for MW181 and MW108) or DMSO (for SB239063). The plate was covered with clear sealing tape and incubated at ambient temperature. After three hours, crystals were retrieved with a 20 micron 0.4 mm CryoLoop (Hampton Research, CA) and flash frozen using liquid nitrogen and 10% (v/v) glycerol as a cryoprotectant. The incubation time was determined by evaluation of the active site occupancy by ligand based on diffraction data. The structure was determined by molecular replacement using the program PHASER and starting model 3 HVC with ligands and waters removed. Iterative model building was performed in Coot, refinement was performed with REFMAC5, and solvent was added using ARP/wARP essentially as previously described [35]. TLS refinement was applied for the final model [36]. Coordinates, structure factors, and details of data collection and processing are available in the protein data bank (www.pdb.org) for the complexes of p38αMAPK with MW181, MW108 and SB239063 under the accession codes 4F9Y, 4F9W and 4FA2, respectively. In evaluating distances between ligand and protein atoms, the NCONT program (CCP4 Program Suite 6.2.0,1994) was used. To measure distances from ring centers to atoms, SYBYL-X v1.3 (Tripos International, Certera St. Louis, MO) was used. A superposition of p38αMAPK complexes was calculated by least squares fitting over all atoms.

### Quantitative Cell-based Activity Screen

The ability of compounds to reduce stressor-induced up-regulation of proinflammatory cytokine production was tested in the murine microglial BV-2 cell line stimulated with LPS as previously described [37]. For experiments, cells were plated in 48-well tissue culture plates at 2×10^4^ cells/well and cultured for 24 hrs. Serum-containing medium was then removed and cells were treated with either saline vehicle control or 100 ng/ml LPS stimulus (LPS from Salmonella enterica serotype typhimurium; EU/mg 600,000; Sigma Cat. No. L6143) in the absence or presence of increasing concentrations of compound, with at least six concentrations of compound ranging from 0.45 µM to 30 µM. Stock solutions of compounds were made in sterile saline (0.9% sodium chloride) that was free of preservatives (Hospira, Inc., Lake Forest, IL: NDC 0409-4888-10). Solutions for cell treatments were prepared by dilution of the stock solutions into serum-free media immediately before adding to the cells. Compound was added to cell cultures just before LPS addition. In some experiments, BV-2 cells were stimulated with other TLR ligands as described previously [37]. TLR ligands used were TLR2∶10 µg/ml LTA, TLR4∶100 ng/ml LPS, TLR7/8∶500 ng/ml CL097, and TLR9∶500 ng/ml ODN1668. Cells were harvested after 18 hrs of treatment for cytokine measurements.

Levels of IL-1β in cell lysates were measured by ELISA using kits from Meso Scale Discovery (MSD; Gaithersburg, MD), as previously described [37]. For the most consistent cytokine measurements with the lowest intra- and interassay variability, cell lysates were frozen at −80°C for at least one hour, then thawed prior to cytokine assay. Cytokine levels were determined by comparison to standard curves ranging from 2.4 pg/mL to 10,000 pg/mL, and confirmation that sample values were on the linear part of the standard curves. The levels of IL-1βwere normalized to the LPS-stimulated vehicle-treated control group for each 48-well tissue culture plate, with data presented as percent of LPS alone. Data represent 3-to-10 independent experiments.

### Cell-based Target Engagement and Activity

The concentration dependent ability of the compounds to inhibit LPS-induced phosphorylation of MK-2, a highly selective p38αMAPK substrate, was examined in BV-2 cells as previously described [37]. BV-2 cells were plated in 48-well tissue culture plates at 2×10^4^ cells/well and treated in serum containing media with LPS in the absence or presence of compounds as described above. After 60 min of treatment, cell lysates were prepared, and phospho-MK-2 levels were measured by MSD ELISA kits. MSD signal was baseline corrected by subtracting the un-stimulated vehicle-treated control group. The data were then normalized to the LPS-stimulated vehicle-treated control group for each 48-well tissue culture plate. Data are presented as percent of LPS alone, and represent 2-to-3 independent experiments.

The loss of inhibitory activity in LPS-induced microglia where the endogenous p38αMAPK gene was disrupted by knock-in of a fully active but drug resistant MAPK mutant, p38α^T106M^ knock-in [38], was tested in primary microglia cultures as previously described [37]. Briefly, mixed glial cultures were prepared from the cerebral cortex of 1–3 day old neonatal C57Bl/6 mice (WT) or p38α^T106M^ knock-in mice, and microglia were isolated from the mixed glial cultures by the shake-off procedure as described [37]. Microglia were stimulated with LPS (3 ng/ml) or vehicle control for 24 hrs in the absence or presence of inhibitors, and IL-1β levels measured as described above. Data are expressed as a percent of the maximal activity, where activity in the presence of LPS alone is taken as 100%. Data represent 3 independent experiments.

### Animal Ethics Statement

All experiments were conducted in accordance with the principles of animal care and experimentation in the Guide For the Care and Use of Laboratory Animals. The Institutional Animal Care and Use Committees of the three universities approved the use of animals in this study. For efficacy experiments, 3- to 4-month-old C57BL/6J male mice from a colony bred in the Columbia University animal facility were used. At the University of Kentucky, the *in vivo* LPS experiment was done with 2-month-old, female, C57Bl/6J mice from Jackson Laboratory. The p38αMAPK drug-resistant knock-in (p38α^T106M^) mice were generated by replacement of Thr106 in p38α with Met as previously described [38]. Remaining rodent studies were done at Northwestern University as previously described [31], [32].

### *In vivo* Efficacy Related to CNS Inflammatory Response

The ability of compounds to inhibit an acute CNS inflammatory response was tested in mice administered LPS to induce brain cytokine production as previously described [37]. Briefly, 2-month old, female, C57Bl/6 mice were administered LPS (0.5 mg/kg) by *i.p.* injection and received compound (20 mg/Kg) or saline vehicle by oral gavage in a volume of 200 µL one hr prior to the LPS injection. At 6 hrs after LPS administration, mice were euthanized, perfused with phosphate-buffered saline (PBS), and brain cortex homogenate supernatants prepared as described [37]. IL-1β levels in 50 µL of supernatant were determined by MSD ELISA. BCA Protein Assay (Pierce) was used to normalize the total amount of protein in the sample loaded.

### *In vivo* Efficacy Related to Attenuation of Synaptic Dysfunction

The ability of compounds to attenuate synaptic dysfunction induced by toxic preparations of human Aβ42 was examined by a hierarchal approach as previously described [39]. The approach includes an initial electrophysiological analysis in brain slices and a subsequent *in vivo* screen using behavioral analyses.

#### Aβ preparation

Aβ42 was prepared as previously described [40], starting from lyophilized peptide (American Peptide) that was suspended in 100% 1,1,1,3,3,3-hexafluoro-2-propanol (Sigma-Aldrich) and allowed to evaporate. The resulting clear peptide film was stored at 20°C. Twenty-four hours prior to its use, the film was suspended in DMSO (Sigma-Aldrich) and sonicated for 10 min. This preparation was diluted into the bath solution, vortexed for 30 sec, and incubated at 4°C for 24 hrs. Western blot analysis was routinely utilized to check the biochemistry of this aged synthetic Aβ.

#### Electrophysiological studies

Brain slices (400 µm) were cut and maintained in an interface chamber at 29°C for 90 min prior to recording, as previously described [40]. CA3-CA1 responses were recorded by means of a stimulating electrode, a bipolar tungsten electrode, placed at the level of the Schaeffer collateral fibers, and a recording electrode, a glass electrode filled with bath solution. Following assessment of basal synaptic transmission by plotting the stimulus voltages against slopes of field EPSP, a 15 min baseline was recorded every minute at an intensity that evokes a response ∼35% of the maximum evoked response. Then, LTP was evoked through a θ-burst stimulation (4 pulses at 100 Hz, with the bursts repeated at 5 Hz and each tetanus including three 10-burst trains separated by 15 sec). In these experiments, 200 nM Aβ42 or vehicle were perfused through the bath solution for 20 min prior to application of the θ-burst, in the absence or presence of 10 µM MW181 or MW108.

#### Behavioral studies

*A)* Reference memory was studied with the 2-day radial arm water maze (RAWM) as described [41]. Aβ42 (200 nM, in a final volume of 1 µl over 1 min) or vehicle were bilaterally infused 20 min prior to the 1st trial (for the 1st group of tests of the RAWM) and 20 min prior to the 7th trial (for the 2nd group of tests of the RAWM), into dorsal hippocampus of mice that had been pre-implanted with a cannula the week before. MW181 or MW108 (5 mg/Kg, *i.p.*) were administered 30 min before the 1st and 2nd group of tests for the RAWM. As controls for these experiments, visible platform testing was conducted to exclude that visual, motor and motivation deficits affect the mouse performance [39]. *B)* Fear conditioning to examine both contextual and cued learning was performed as previously described [42]. Aβ42 was infused as above, 20 min prior to the foot shock. Compounds were administered before training for fear conditioning. For these experiments, threshold assessment test was performed to check sensory perception of electric shock in different groups of mice, as previously described [42]. In addition, the open-field test was conducted to evaluate exploratory behavior, as previously described [43].

### Statistics

For activity screens, statistical analysis was conducted using GraphPad prism software (GraphPad Software, San Diego California USA). Calculations of IC_50_ values were made using a nonlinear regression with a variable Hill slope, with the data normalized to the positive control to fit the top and bottom plateaus. Values are expressed as mean ± SEM. Groups of 2 were compared by unpaired T-test. Groups of 3 or more were compared by One-way analysis of variance (ANOVA), followed by Bonferroni Multiple Comparison Test. For efficacy studies, the experiments were performed in blind (results expressed as mean ± SEM). Significance of differences between 2 groups was determined by t-test for pairwise comparisons or a 2-way ANOVA with repeated measures for multiple comparisons. In all experiments, significance was set at p<0.05.

## Results

### Design Strategy for Synthetic Refinement of p38αMAPK Inhibitor Hit

The isoform-selective p38αMAPK inhibitor MW01-11-108 (MW108) and the p38MAPK subfamily inhibitor MW01-10-181SRM (MW181) reported here were developed by structure- and pharmacoinformatics-driven synthetic refinement of an initial p38αMAPK inhibitor hit [31], MW01-2-069A-SRM (MW069a). MW069a is a positional isomer of MW01-6-189WH [32], also referred to as TT301 in clinical studies. While MW01-6-189WH does not interact with p38MAPKs, MW069a binds to the active site, as demonstrated by high-resolution co-crystallography (PDB 4EWQ), and is a novel, brain penetrant, p38MAPK inhibitor that exhibits *in vivo* function in an AD mouse model [31]. However, the MW069a hit had secondary kinase activity with p38βMAPK and CK1δ similar to other existing p38MAPK inhibitor reagents and drugs in clinical development. Therefore, we used the high-resolution crystal structure of 4EWQ as the starting point for ligand design that was coupled with pharmacoinformatics considerations to restrict syntheses to analogs with CNS drug-like properties and low potential for CYP2D6 substrate status [6], [7].

### Design, Synthesis and Chemical Properties of Novel and Selective p38αMAPK Inhibitors Targeting the Active Site

**Figure 2:**
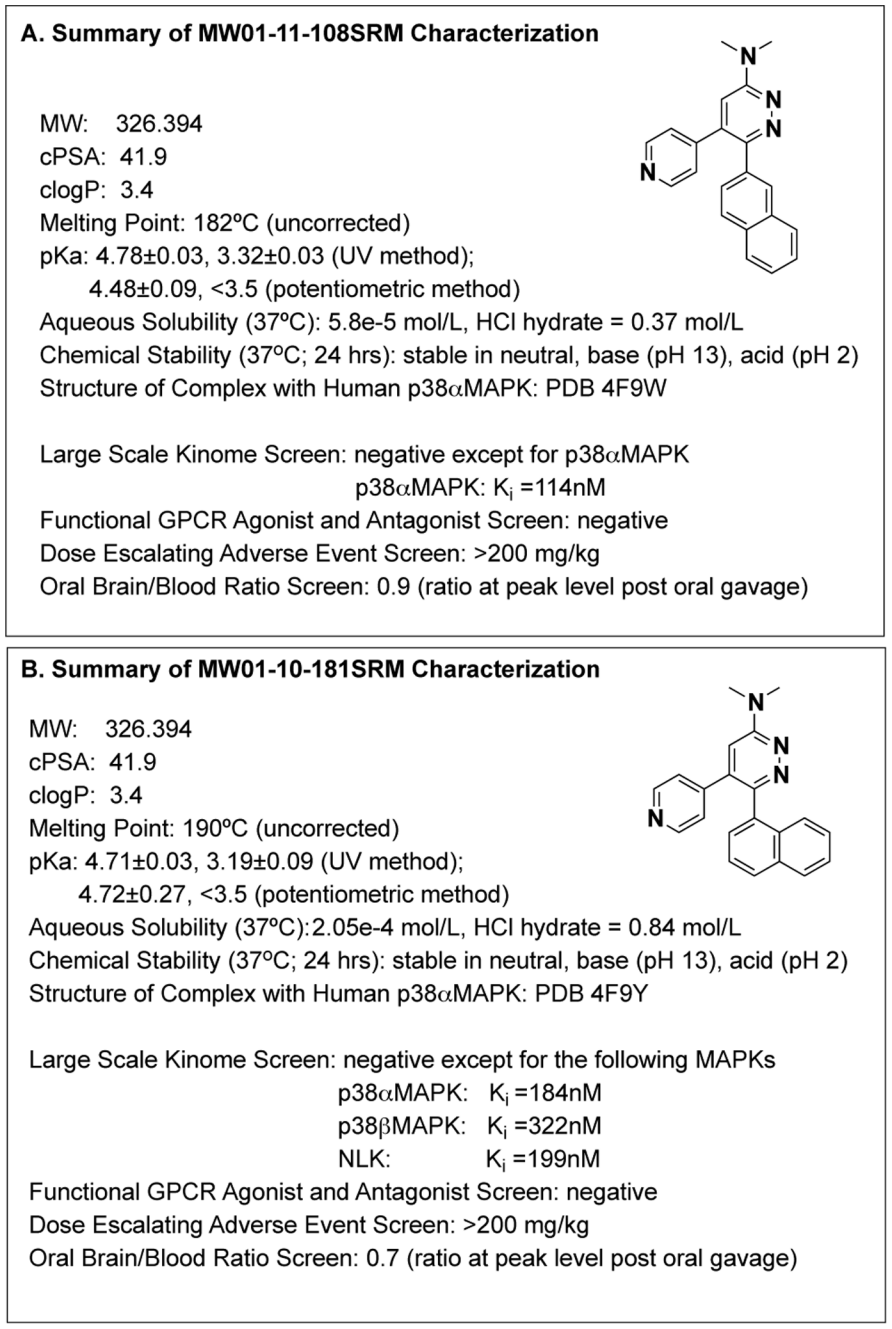
Characterization of selective p38αMAPK inhibitors MW108 (panel A) and MW181 (panel B). The title compounds were characterized in terms of their physical characteristics, target selectivity, and pharmacological properties.

The design, synthesis and molecular characterization of the MW108 and MW181 title compounds reported here are summarized in Materials and Methods, Results, **Figures 2** **and** **3** **, Scheme S1, and Figure S1**. The novel, brain penetrant inhibitors are soluble, acidic, and chemically stable small molecules targeted to the active site of p38αMAPK.

**Figure 3:**
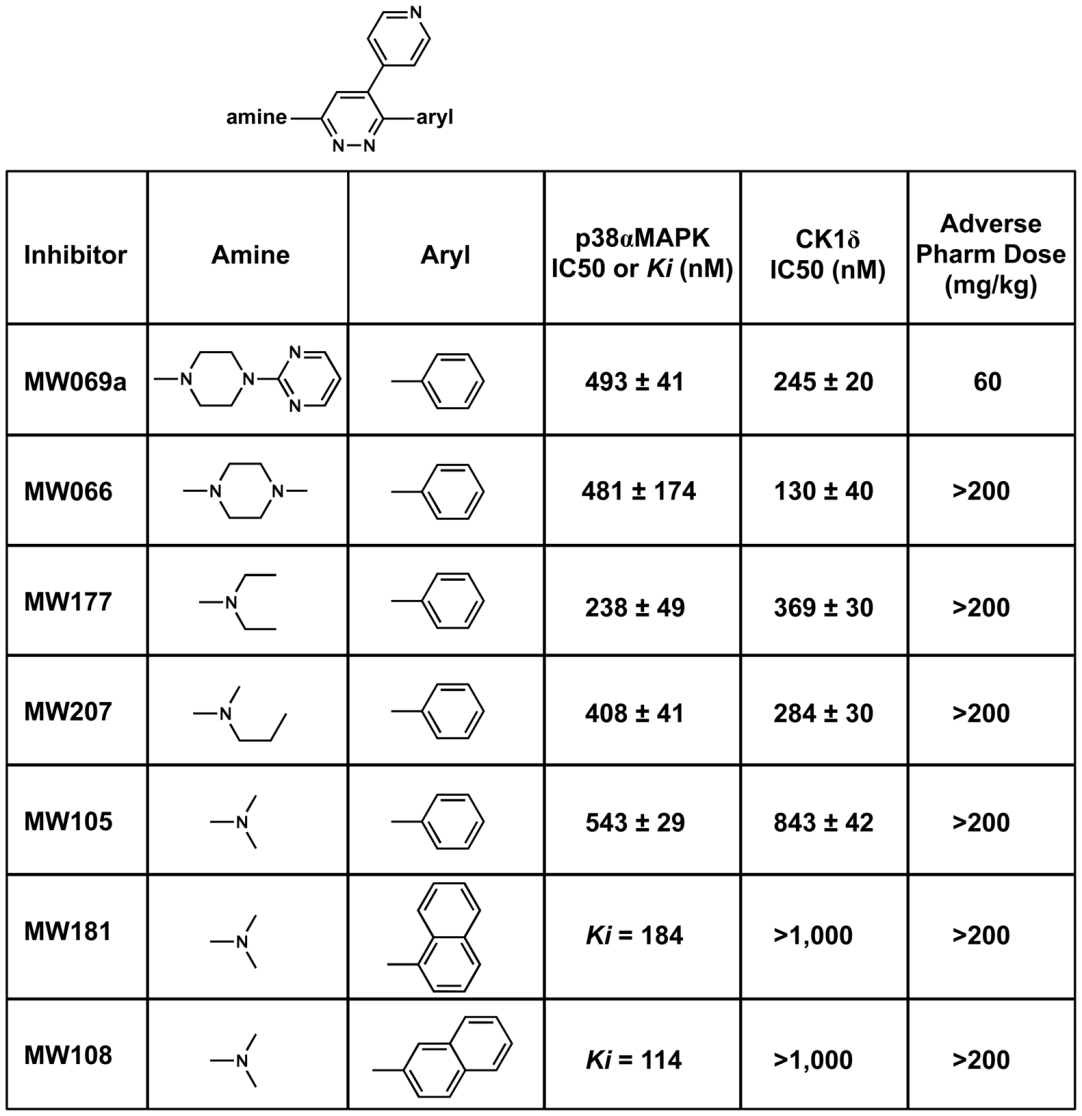
Synthesis of p38αMAPK inhibitor analogs and initial screens for differential p38αMAPK vs CK1δ inhibition and for improvement in high dose tolerance. The affinity of compounds for p38αMAPK and CK1δ is shown, as well as the highest dose at which no adverse events are observed in dose escalation studies.

As is evident from **Figure 2**, the inhibitor structures are rule-of-four compliant (i.e., MW<400, clogP<4, PSA<80Å^2^), a multi-property feature characteristic of most blood-brain barrier penetrant small molecule drugs and *in vivo* probes [6], [7]. The experimental pKa determination values derive from two different end points and reveal that the two inhibitors are acidic, consistent with computed values of 3.06±0.10 and the known properties of modified pyridazines [44]. The experimental values for aqueous solubility bracket the computed value of 4.9e-5 mol/L (pH 7.0) for both structures. The commonly used HCl hydrate formulations increase aqueous solubility significantly to ∼ 0.4 and 0.8 mol/L for MW108 and MW181, respectively. The results document that the particular multi-aromatic system used in these chemical probes is not likely to generate technical issues related to performance in assays, and that the HCl hydrate form preferred for *in vivo* studies will be readily soluble in aqueous buffers or physiological saline solutions. Overall, the experimental results on physical properties are consistent with the small molecule structures built around the privileged pyridazine core used in clinical therapeutics and preclinical *in vivo* research reagents.

The campaign started with a hit, MW01-2-069A-SRM, that is a p38αMAPK inhibitor with *in vivo* CNS function. It emerged from a structure-assisted, scaffold-reutilization design study based on a clinically safe drug [31]. The co-crystal structure of the hit in complex with human p38αMAPK (PDB 4EWQ) provided the structure-based rationale for the ligand design (see **Figure S1** for diagram of design strategy). Previous synthesis and testing [31] revealed a critical need for the pyridine substituent with its ring nitrogen at the appropriate position and the need for an aryl substituent at the R6 position on the pyridazine ring. Ligand designs were filtered by pharmacoinformatics criteria based on prior success in CNS drug discovery, with a focus on key physical properties as a filter [6], [7].

Several biology-relevant outcomes emerged during development (**Figure 3**). Improvement of the no adverse effects levels (NOAEL, the highest dose at which no adverse events are observed in dose escalation studies) in mice emerged from simplification of the amine at the R3 position of the pyridazine core (e.g., inhibitors MW066, MW177, MW207, MW105), while still retaining cross-over kinase inhibition. This is consistent with neither the multi-kinase nature of inhibitors nor the target itself being a major contributor to adverse events. While the reduced size of the substituent at the R3 position improved pharmacological properties in the *in vivo* screen, it resulted in loss of potential ligand-protein interactions in this region of the structure. However, the retention of p38αMAPK activity and acquisition of improved safety with simplification of the R3 amine (as in MW108 and MW181) allowed introduction of bulkier naphthyl substituents at the R6 position, to better fill the hydrophobic pocket, while remaining compliant with rule of 4 guidelines for CNS penetrance [6], [7]. The introduction of the 2-naphthyl (MW108) or 1-naphthyl (MW181) substituent at R_6_ on the pyridazine core was key to improvement of p38αMAPK affinity and concomitant loss of CK1δ affinity. Further, the presence of a 2-naphthyl in MW108 vs a 1-naphthyl in MW181 also resulted in a loss of affinity for p38βMAPK and NLK with retention of p38αMAPK affinity.

### Kinase Profiling and Off-target Screens for Selectivity of MW108 and MW181

Large-scale kinome screens of MW108 revealed only p38αMAPK as a potential target (see **Table S1**). Follow-up enzyme kinetics demonstrated a p38αMAPK Ki value of 114 nM for MW108 (**Figure 3** **,** **Figure S2**). Large-scale kinome screens of MW181 revealed p38αMAPK, p38βMAPK, and the atypical p38 kinase NLK as primary targets (see **Table S2**), with subsequent kinetic analyses yielding Ki values, respectively, of 184 nM (**Figure 3** **,** **Figure S3**), 322 nM and 199 nM. Noteworthy overall is the lack of CK1δ inhibition by either MW108 or MW181, and the increased selectivity of MW108 for the p38αMAPK isoform. In terms of the potential for unanticipated off-target functions, both MW108 and MW181 were negative in functional GPCR agonist and antagonist screens, and neither compound revealed adverse pharmacology in mice at the highest dose tested (**Figure 3**; 200 mg/kg, 10–40 fold higher than *in vivo* doses used in efficacy screening assays). Both compounds were CNS penetrant, as assessed by the brain/blood peak ratio after oral administration. This composite profile qualified the novel inhibitors for further biological and structural analyses.

### High-resolution Co-crystal Structures of Compounds with Human p38αMAPK

**Figure 4:**
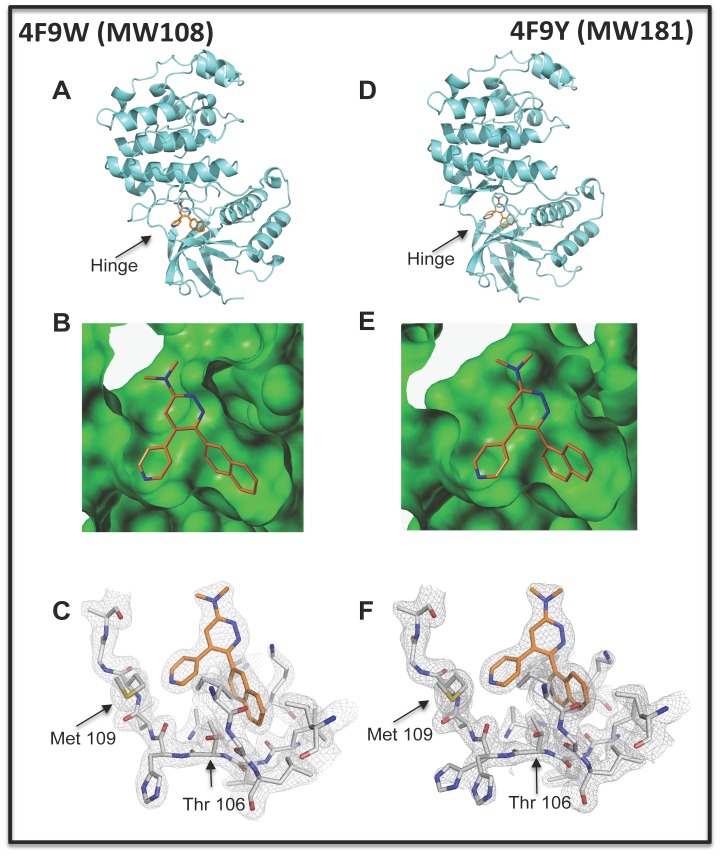
Crystal structures of human p38αMAPK with MW108 (PDB 4F9W) or MW181 (PDB 4F9Y) bound in the active site. **A.** The 2.00Å structure (4F9W) of human p38αMAPK (cyan ribbons) containing MW108 (colored by atom type) bound in the active site is shown. The typical kinase bi-lobed nature is evident, with the hinge region connecting the two lobes of the kinase indicated by the arrow. **B.** A Connolly surface generated from the coordinates of 4F9W highlights the complementary features between MW108 and the kinase active site. **C.** Electron density map (2mFo-Dfc) for 4F9W contoured at a 1.5 rmsd level. The diffraction data clearly reveals the inhibitor presence in the active site. The relative positions of Met 109 in the hinge region and the hydrophobic pocket’s small gatekeeper residue Thr 106 are indicated by arrows. The pyridine ring nitrogen of MW108 makes H-bond interactions with the protein backbone at the Met109-Gly110 peptide bond. **D.** The 1.85Å resolution structure (4F9Y) of human p38αMAPK (cyan ribbons) with MW181 (colored by atom type) bound in the active site is shown, with the hinge region indicated by the arrow. **E.** A Connolly surface generated from the coordinates of 4F9Y highlights the complementary features between MW181 and the kinase active site. **F.** Electron density map (2mF0-Dfc) for 4F9Y contoured at 1.5 rmsd. The difference in orientation of the1-naphthyl substituent in MW181 4F9Y from that of the 2-naphthyl substituent in MW108 4F9W (Panel C) is evident from the diffraction data.

To confirm the active site targeting of MW108 and MW181 and test prevailing hypotheses, we determined the high-resolution co-crystal structures of human p38αMAPK in complex with the two selective inhibitors. As summarized in **Figure 4**, both inhibitors occupy the active site, engage the hinge region peptide backbone at Met109, and occupy the nearby hydrophobic pocket. The structures and the associated crystallographic data are deposited and available in the Protein Data Bank under accession numbers 4F9W (containing MW108) and 4F9Y (containing MW181). A summary of key diffraction data fitting statistics is provided in **Table 1**. Both complexes clearly exhibit the DFG-in motif notation used widely in protein kinase conformation analyses, although this is no longer used as a conformational change that correlates with or forecasts inhibitor activity [15]. Importantly, neither compound induced a hinge region glycine flip.

**Table 1:** Data collection and phasing statistics. Values in parentheses are for highest resolution shell.

	4F9W(MAPK-108)	4F9Y(MAPK-181)	4FA2(MAPK-SB239063)
**Data Collection**			
Space group	P2_1_2_1_2_1_	P2_1_2_1_2_1_	P2_1_2_1_2_1_
Wavelength (Å)	0.97872	0.97856	0.97872
Cell dimensions *a,b,c* (Å)	65.6, 74.3, 77.2	66.5, 74.8, 78.2	65.1, 74.1, 77.7
Resolution (Å)	30.00–2.00	50.00–1.85	30.00–2.00
Outer Resolution Shell (Å)	(2.03–2.00)	(1.88–1.85)	(2.03–2.00)
Completeness (%)	99.9 (99.9)	99.9 (99.9)	99.9 (100.0)
R_merge_	0.075 (0.678)	0.039 (0.537)	0.059 (0.440)
Mean I/σ(I)	24.5 (2.5)	42.4 (3.8)	30.8 (4.8)
Redundancy	6.1 (5.7)	7.3 (7.3)	7.3 (7.4)
No. of Unique Reflections	26189	34190	26070
**Refinement statistics**			
Resolution (Å)	29.80–2.00	33.76–1.85	29.81–2.00
R_work_	0.17 (0.22)	0.16 (0.21)	0.17 (0.18)
R_free_	0.21 (0.26)	0.20 (0.26)	0.21 (0.25)
Residues	336	339	339
Atoms			
Protein	2769	2913	2873
Ligand	25	25	27
Waters	186	314	185
Mean B-value (Å^2^)			
Overall	35.1	29.0	31.1
Protein	40.6	36.6	38.4
Ligand	32.2	34.9	30.6
Waters	45.1	43.5	45.0
RMSD bond lengths (Å)	0.012	0.012	0.012
RMSD bond angles (^o^)	1.50	1.36	1.33
Ramachandran plot, %			
Most Favored	97.6	97.3	97.9
Allowed	2.1	2.4	2.1
Generously Allowed	0.3	0.3	0.0
Disallowed	0.0	0.0	0.0

**Figure 5:**
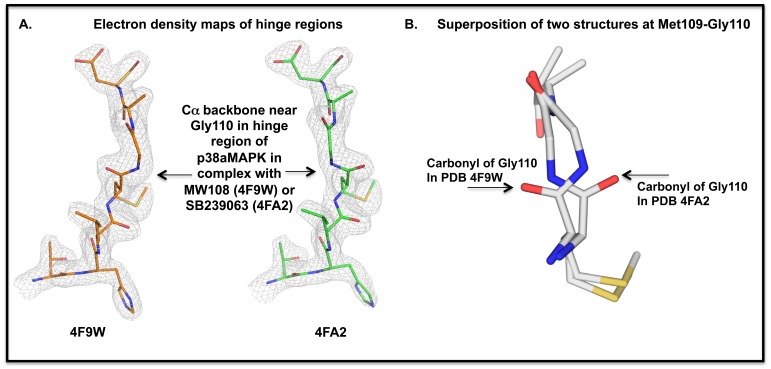
Localized conformational change in the hinge region of human p38αMAPK when a non-selective inhibitor is bound. **A.** Electron density maps (2mFo-Dfc; colored in light gray) contoured at 1.5 rmsd for the hinge region of p38αMAPK near Gly110 for 4F9W (in complex with MW108) and for 4FA2 (in complex with SB239063) are shown. Differences in the electron density path are seen in the area around Gly110, indicated by arrows. **B.** A superposition of 4F9W and 4FA2 reveals the differences around the peptide bond at Met109-Gly110, referred to as a glycine flip for structures such as 4FA2.

The high-resolution co-crystallography results raised the question of why we were able to generate such a selective inhibitor that lacked a localized conformational change assumed to be a key factor in generating selectivity [16]–[30]. To address if our experimental protocol could detect such localized conformation changes, we performed a control crystallographic study of p38αMAPK in complex with SB239063. SB239063 is representative of a widely used class of p38αMAPK inhibitors that are mixed kinase inhibitors, with activity against both CK1δ and p38MAPKs. The crystallization and formation of the complex as well as data collection and processing were done as performed for the p38αMAPK complexes with MW108 and MW181. The resultant structure and the associated crystallographic data for the human p38αMAPK in complex with SB239063 are available in the Protein Data Bank under accession identifier 4FA2. Superposition analyses using least squares fitting revealed conformational similarity among the three structures (4F9W, 4F9Y, 4FA2) except for the deviation of 4FA2 from the other two conformations in the hinge region of the kinase. The well-defined crystallographic data in the hinge region demonstrates a difference in the peptide bond between Met 109 and Gly 110 in the 4FA2 complex compared to 4F9W and 4F9Y (**Figure 5**). Specifically, the alpha carbon (Cα) of Gly 110 differs by 1.89 Å and the carbonyl oxygen of Met 109 is displaced by 2.81 Å. This observation correlates with repositioning of the protein backbone conformation referred to as the glycine-flip. The control 4FA2 results demonstrate that such a conformational change can be detected in human p38αMAPK by the methods used for 4F9W and 4F9Y, but these latter structures do not manifest the alternative conformation. Further, the results with the control 4FA2 complex demonstrate that the glycine flip conformational change in p38αMAPK does not correlate with selectivity for p38αMAPK.

### CNS Mechanism of Action and Linkage to a Pharmacodynamic End Point

**Figure 6:**
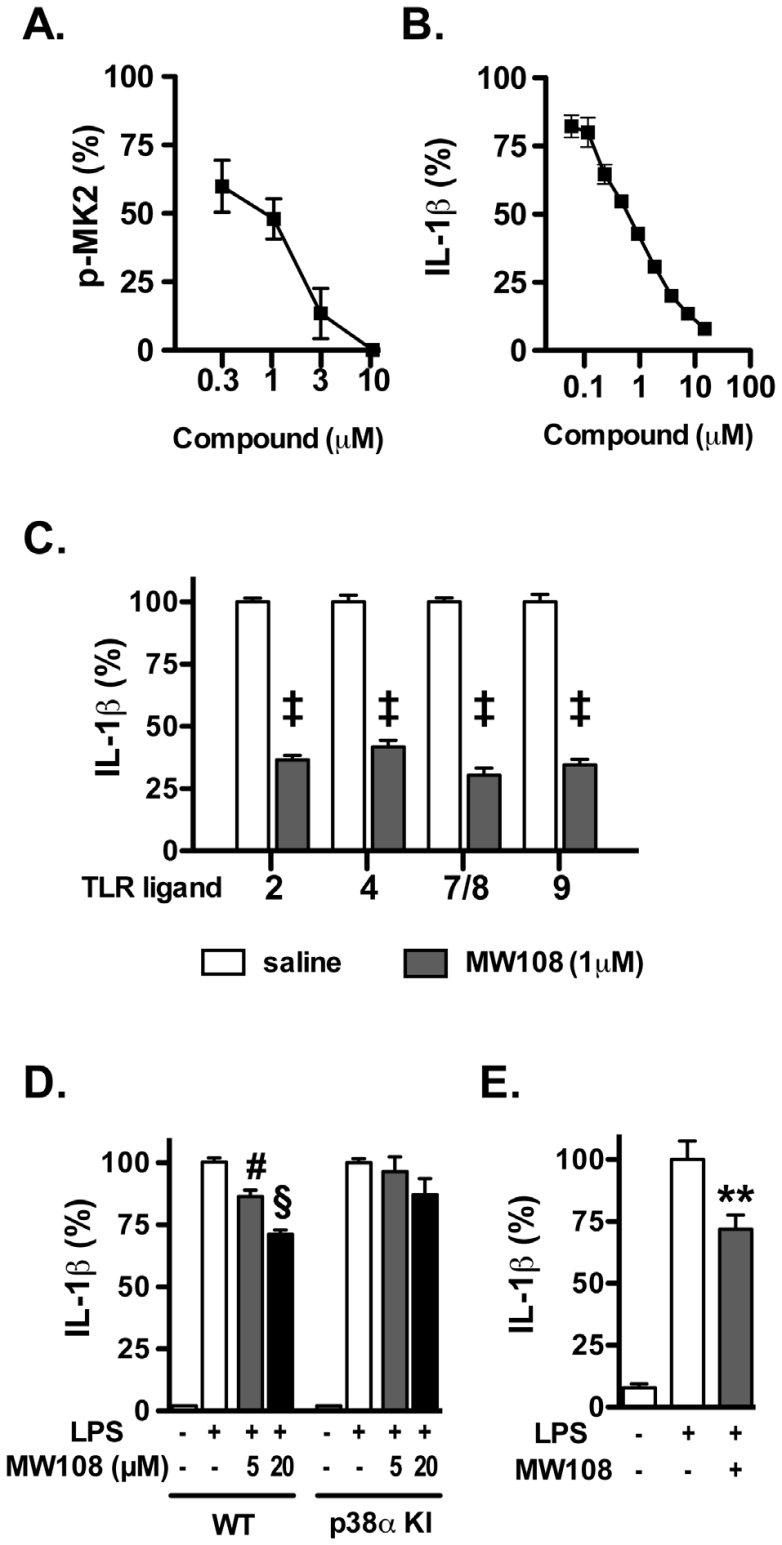
Cellular target engagement and mechanism of action of MW108. **A.** MW108 inhibited phosphorylation of the p38α substrate MK2 in a concentration-dependent manner in LPS-stimulated BV-2 microglial cells. **B.** MW108 inhibited LPS-induced IL-1β production in a concentration-dependent manner in BV-2 cells. **C.** MW108 suppressed IL-1β production in BV-2 cells stimulated with diverse TLR ligands. BV-2 cells were treated with ligands for TLR2, TLR4, TLR7/8, and TLR9 in the absence (white bars) or presence (gray bars) of MW108. ‡ *p*<0.0001 compared to TLR ligand in the absence of compound. **D.** MW108 inhibited LPS-induced IL-1β production in primary microglia from wild-type (WT) mice, but not from drug-resistant p38αMAPK knock-in (p38α KI) mice. # *p*<0.01, § *p*<0.001 compared to LPS alone. **E.** MW108 suppressed LPS-induced IL-1β levels *in vivo*. **p<0.01 compared to LPS alone (n = 6 saline/vehicle; n = 24 LPS/vehicle; n = 15 LPS/MW108). Data in all panels are expressed as a percent of the maximal levels, where levels in the presence of stressor alone were normalized to 100%.

The ability of MW108 to engage its target in a cellular assay was done by examining the concentration-dependent inhibition of phosphorylated MK-2, considered a highly selective p38αMAPK substrate. **Figure 6A** shows the results with the microglial cell line BV-2 stimulated with the standard glial activating stimulus lipopolysaccharide (LPS). There is a clear decrease in phosphorylated MK-2 in LPS-stimulated cells with increasing concentration of MW108. LPS activation of the signal transduction pathway involving p38αMAPK and its substrate MK-2 results in a downstream increase in proinflammatory cytokine production. Therefore, we quantified the levels of the proinflammatory cytokine interleukin 1β (IL-1β) as a function of MW108 concentration (**Figure 6B**), and showed a coincident decrease over a similar concentration range. The IC_50_ using inhibition of IL-1β overproduction as an end point was 610±20 nM.

To extend the general biological relevance of the initial cellular engagement studies, we examined the ability of MW108 to inhibit increases in IL-1β levels using different stressors, wild-type and inhibitor resistant primary cells, and *in vivo* responses. As shown in **Figure 6C**, the inhibition of IL-1β production is not limited to LPS as a stressor, but was also seen in BV-2 cells stimulated with diverse TLR ligands (TLR2, TLR4, TLR7/8, TLR9). As shown in **Figure 6D**, further validation was provided by demonstrating a loss of MW108 activity in primary microglia cultures where the endogenous p38αMAPK was removed by targeted knock-in replacement with a fully active but drug resistant mutant p38α^T106M^MAPK. The knock-in yields catalytically normal kinase, but the replacement of the gatekeeper Thr at residue 106 with a larger side chain Met amino acid renders the mutant p38α^T106M^MAPK resistant to inhibitors such as MW108 that exploit the use of the hydrophobic pocket with bulky substituents such as a naphthyl group. To probe the *in vivo* relevance of the proinflammatory cytokine modulation seen in glia cultures, we tested if oral administration of MW108 could attenuate stressor induced IL-1β increases in the brain. As shown in **Figure 6E**, oral administration of MW108 restored IL-1β levels in the brain cortex back towards control. Altogether, these results demonstrate that MW108 engages its molecular target p38αMAPK and modulates the linked cellular response of increased proinflammatory cytokine production, and that this target-related function is evident *in vivo*.

**Figure 7:**
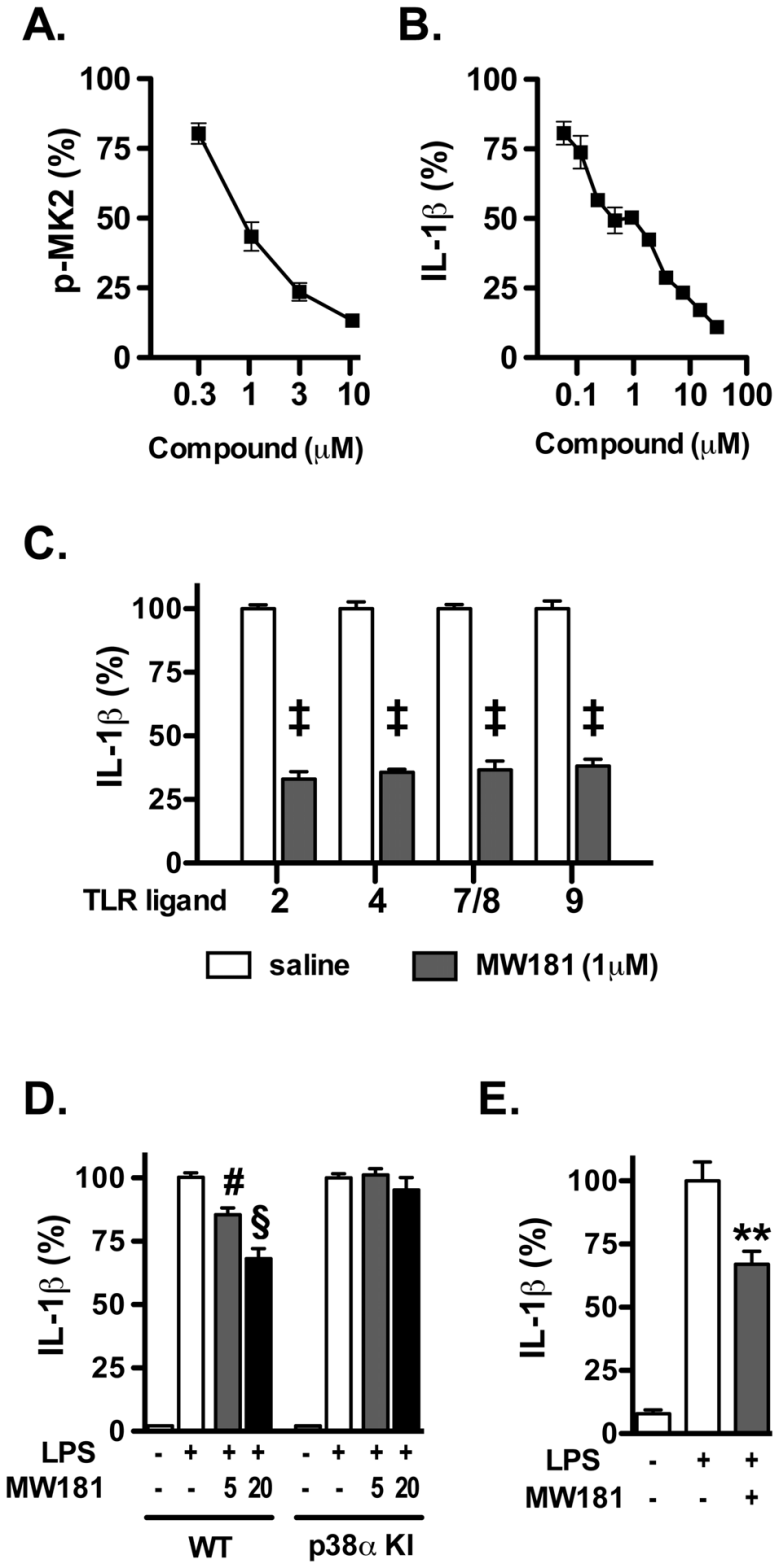
Cellular target engagement and mechanism of action of MW181. **A.** MW181 inhibited phosphorylation of the p38αMAPK substrate MK2 in a concentration-dependent manner in LPS-stimulated BV-2 microglial cells. **B.** MW181 inhibited LPS-induced IL-1β production in a concentration-dependent manner in BV-2 cells. **C.** MW-181 suppressed IL-1β production in BV-2 cells stimulated with diverse TLR ligands. BV-2 cells were treated with ligands for TLR2, TLR4, TLR7/8, and TLR9 in the absence (white bars) or presence (gray bars) of MW181. ‡ *p*<0.0001 compared to TLR ligand in the absence of compound. **D.** MW181 inhibited LPS-induced IL-1β production in primary microglia from wild-type (WT) mice, but not from drug-resistant p38αMAPK knock-in (p38α KI) mice. # *p*<0.01, § *p*<0.001 compared to LPS alone. **E.** MW181 suppressed LPS-induced IL-1β levels *in vivo*. **p<0.01 compared to LPS alone (n = 6 saline/vehicle; n = 24 LPS/vehicle; n = 15 LPS/MW181). Data in all panels are expressed as a percent of the maximal levels, where levels in the presence of stressor alone were normalized to 100%.

A prevailing hypothesis in kinase inhibitor development based on analysis of successful drug development campaigns is that *in vivo* efficacious kinase inhibitors engage a close set of multiple kinases [15]. Relatedly, many existing p38αMAPK inhibitors are equivalent or better inhibitors of other p38MAPK family members (e.g., p38βMAPK and NLK) or unrelated kinases with convergent active site folds (e.g., CK1). To address whether a p38MAPK inhibitor that inhibits other p38MAPK family members, but lacks CK1 inhibition, would have cellular activities similar to MW108, we examined the quantitative activity of MW181. MW181 exhibited activities similar to MW108, including inhibition of increased cytokine production by glia in response to LPS (IC_50_ = 820±30 nM) and other TLR ligands, inhibition of MK-2 phosphorylation, loss of activity in drug resistant microglia, and *in vivo* suppression of increased brain levels of IL-1β after oral administraton **(** **Figure 7** **)**. The data indicate that the activity and function of the p38MAPK family inhibitor, MW181, probably reflects its p38αMAPK inhibition in these assays.

### *In vivo* Efficacy following Amyloid-beta Elevation

We have previously documented in multiple AD-relevant animal models [31], [45]–[47] that proinflammatory cytokine overproduction is an early event in the progression of AD pathophysiology that is causally linked to synaptic dysfunction and cognitive deficits. In addition, p38αMAPK has been implicated as a contributor to AD-relevant pathology progression [4]. However, we are not aware of an *in vivo* assay precedent for a single kinase inhibitor being functional in such a CNS disorder model. Therefore, we used a hierarchal evaluation of whether MW108 could attenuate synaptic dysfunction and cognitive impairments.

**Figure 8:**
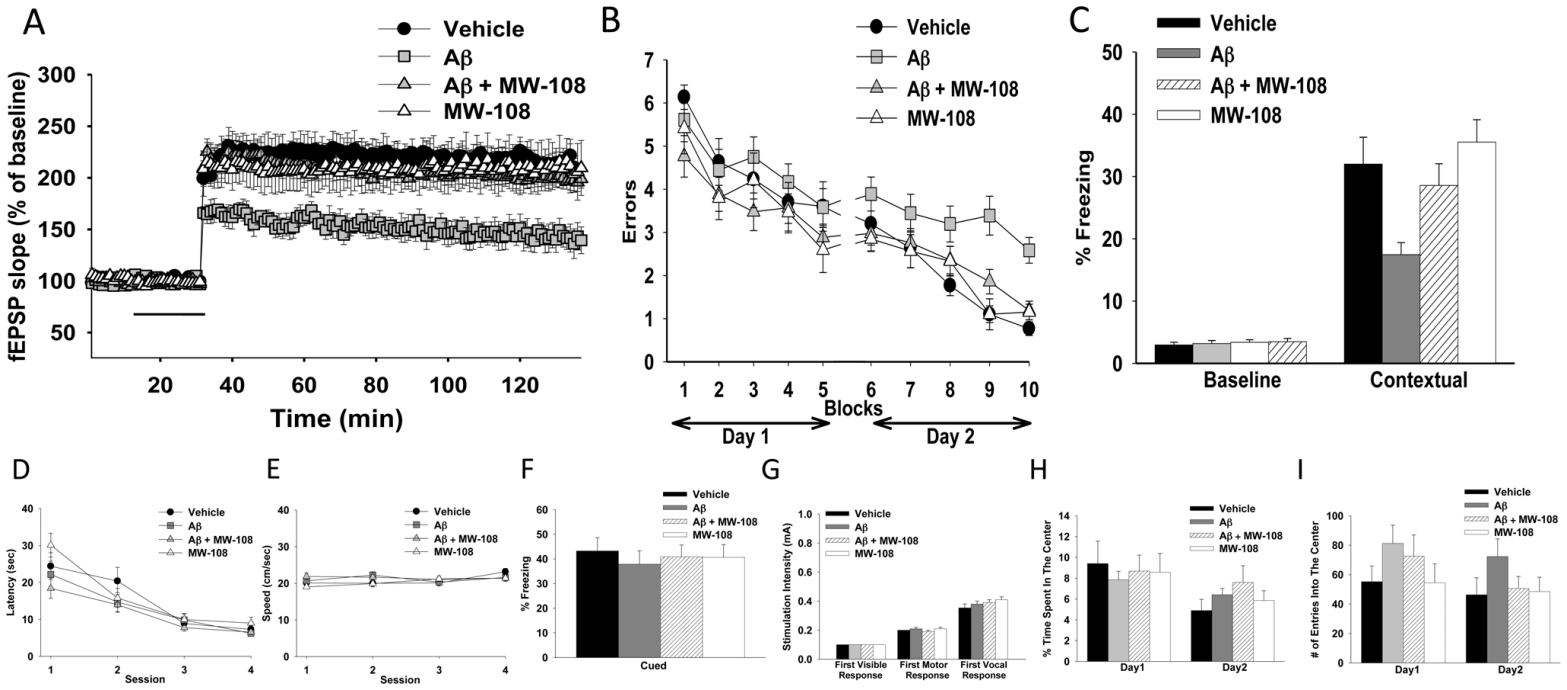
Attenuation of Aβ_42_-induced synaptic and cognitive dysfunction by MW108 administration. **A.** MW108 ameliorated the LTP deficit in Aβ_42_-treated slices (n = 7–8 slices/group; F(1,13) = 17.25, p = 0.0011, compared to slices treated only with Aβ_42_). Horizontal bar = time of application of Aβ_42_ and MW108. **B.** MW108 ameliorated the reference memory deficit in Aβ_42_-infused mice (n = 10–14 mice/group; F(1,24) = 1.827, p = 0.0001 comparing Aβ-infused vs. Aβ-infused+compound). **C.** MW108 ameliorated the contextual fear memory deficit in Aβ_42_-infused mice (n = 16-18 mice/group; p = 0.009 comparing Aβ-infused vs. Aβ-infused+compound). **D-E.** No difference was detected between groups when tested for visual-motor-motivational deficits with the visible platform test; speed and time to the platform are shown in D and E, respectively (n = 10–14 mice/group). **F.** The same animals that underwent contextual fear conditioning testing were assessed for cued learning 24 hrs after the contextual learning. No difference was detected between groups (n = 16–18 mice/group). **G.** Sensory threshold was not affected regardless of treatment (n = 13–17 mice/group). **H-I.** No differences in exploratory behavior, as shown by a similar percentage of time spent in the center compartment (H) and the number of entries into the center compartment (I), were observed (n = 13–17 mice/group).

To assess synaptic function, we used the widely studied long-term potentiation (LTP) experimental model of synaptic plasticity. We induced LTP in the presence of oligomeric Aβ_42_, a peptide that accumulates in the brains of AD patients. As expected from previous studies [39]–[42], [48], Aβ reduced LTP (**Figure 8A**). Importantly, MW108 ameliorated the Aβ-induced electrophysiological deficit (**Figure 8A**), indicative of rescue of synaptic plasticity impairment.

The initial results were extended by testing whether MW108 could attenuate cognitive deficits in two AD-relevant behavioral tasks that measure two types of memory dysfunction: the radial arm water maze (RAWM) to measure reference memory [41] and fear conditioning to measure contextual fear memory [42]. Aβ_42_ or vehicle was bilaterally infused into the dorsal hippocampus of mice. Aβ-infused mice showed memory deficits, exhibiting more performance errors compared to vehicle-infused mice (**Figure 8B**). Treatment with MW108 rescued the memory defects (**Figure 8B**). In the fear conditioning task, Aβ_42_ caused a contextual fear memory deficit which was prevented by MW108 administration (**Figure 8C** **)**. Control experiments, including visible platform test, open field, sensory threshold assessment and cued learning, showed no differences across groups (**Figure 8D-I**). These data document that MW108 attenuates AD-relevant impairments in synaptic plasticity and memory.

**Figure 9:**
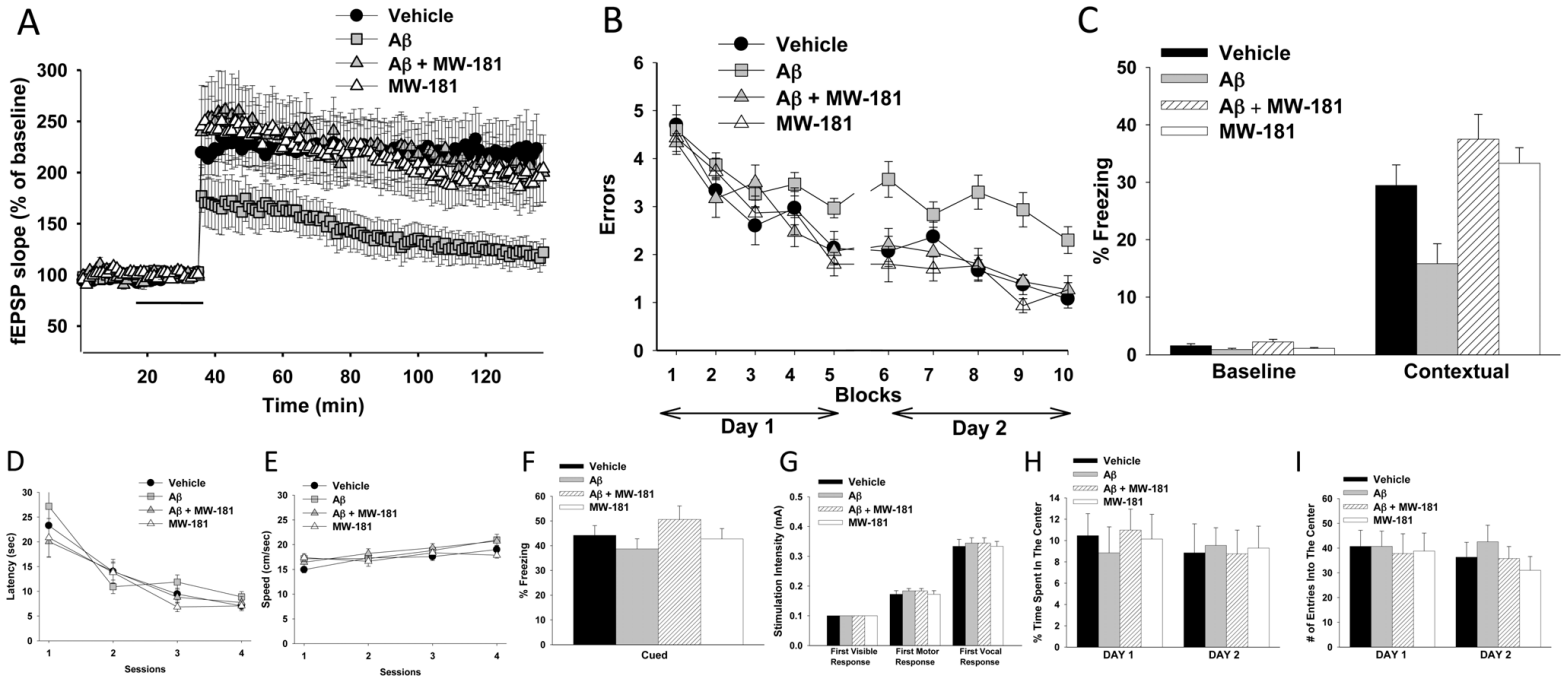
Attenuation of Aβ_42_-induced synaptic and cognitive dysfunction by MW181 administration. **A.** MW181 ameliorated the LTP deficit in Aβ_42_-treated slices (n = 7–8 slices/group; F(1,13) = 6.651, p = 0.0229, compared to slices treated only with Aβ_42_). Horizontal bar = time of application of Aβ_42_ and MW181. **B.** MW181 ameliorated the reference memory deficit in Aβ_42_-infused mice (n = 10 mice/group; F(1,18) = 5.851, p = 0.0264 comparing Aβ-infused vs. Aβ-infused+compound). **C.** MW-181 ameliorated the contextual fear memory deficit in Aβ_42_-infused mice (n = 15–16 mice/group; p = 0.0005 comparing Aβ-infused vs. Aβ-infused+compound). **D-E.** No difference was detected between groups when tested for visual-motor-motivational deficits with the visible platform test; speed and time to the platform are shown in D and E, respectively (n = 10 mice/group). **F.** The same animals that underwent contextual fear conditioning testing were assessed for cued learning 24 hrs after the contextual learning. No difference was detected between groups (n = 15–16 mice/group). **G.** Sensory threshold was not affected regardless of treatment (n = 9 mice/group). **H-I.** No differences in exploratory behavior, as shown by a similar percentage of time spent in the center compartment (H) and the number of entries into the center compartment (I), were observed (n = 9 mice/group).

Similar results were obtained with MW181. Specifically, MW181 was efficacious in rescuing the LTP deficit and the behavioral impairments in RAWM and fear conditioning tasks, with no differences across groups seen in the control experiments (**Figure 9**). Overall, the data document that an isoform selective p38αMAPK inhibitor is efficacious at reducing AD-relevant synaptic and functional impairments, and that mixed p38MAPK family member inhibitors that lack CK1δ and other cross-over inhibitor activities can, under appropriate conditions, be surrogates for the isoform selective inhibitor.

## Discussion

The studies reported here provide precedents and insight for targeting serine-threonine kinases for CNS investigations, and demonstrate the potential of brain p38αMAPK as a target for modulating progression of synaptic dysfunction. First, we demonstrate that targeting the active site ATP-fold of protein kinases is a more viable approach for the discovery of highly selective kinase inhibitors with *in vivo* functional utility than is generally assumed. Our findings provide insight that should be useful in future kinase inhibitor design, and raise caution about exuberance over hypotheses driven by observations on localized conformational changes that do not correlate with experimental data from kinase inhibitor activity profiles. Second, the results demonstrate that an isoform-selective inhibitor of a CNS kinase target can be efficacious *in vivo*. This revises the assumption that *in vivo* efficacy requires appropriate targeting of multiple discrete kinases. Third, adverse pharmacology did not correlate with inhibitor cross-over to other p38MAPK isoforms or to the distinct CK1δ kinase. MW108, MW181, and the intermediate inhibitors generated during refinement all lack frank adverse pharmacology at higher doses observed with MW069a, yet they vary in their kinase inhibitor selectivity among p38MAPK isoforms and CK1δ. Fourth, the combination of cellular and *in vivo* observations reported here implicate the p38αMAPK isoform as a quantitative contributor to CNS stress-related responses and as a molecular target whose inhibition can attenuate synaptic dysfunction. As a potential CNS target, p38αMAPK is especially attractive due to its key roles in both glial and neuronal processes, offering the potential for a more robust *in vivo* inhibition under certain stress related responses. Currently, there are no selective p38αMAPK inhibitors in CNS clinical trials. However, the impact on attenuation of synaptic dysfunction suggests the potential of this kinase as a potential drug discovery target. While the novel *in vivo* chemical probes described here are not drugs or drug candidates, their drug-like properties and pharmacodynamics make them logical starting points for drug discovery studies that must be done to optimize structures and meet FDA recommendations and standard practices in preclinical drug development. Their current biological utility is predominately as small molecule neuroscience research tools to address essential issues, such as biological time windows for effective intervention, and the extension of *in vivo* pathology progression investigations to other animal species.

The high-resolution crystal structures of human p38αMAPK with either MW108 (4F9W) or MW181 (4F9Y) bound in the active site confirm the design features of the discovery platform and provide a starting point for future explorations of the molecular basis of small molecule recognition by p38αMAPK and its extension to other kinase-chemical probe paradigms. For example, the chemical probes and structural insights reported here provide an immediate starting point for exploration of the high-resolution co-crystal structures of active site-directed ligands with greater selectivity for p38βMAPK or CK1δ in order to understand the molecular basis of selective ligand-kinase target recognition between these two branches of the kinome and, by extension, to related protein folds emerging from structural genomics investigations.

While our results are not consistent with kinase selectivity being correlated with a localized induction of a glycine flip conformational change, it is possible for the core pyridazine ring to participate in such an interaction. The pyridazine ring differs from other diazines, such as pyrimidines, due to the different atomic environment provided by two contiguous nitrogen atoms in an aromatic ring and is less acidic in nature, thereby allowing different roles for the pyridazine ring nitrogens depending on the its molecular context in a scaffold [49]. For example, both of the pyridazine ring nitrogens have the potential to engage in H-bond interactions with the Met109-Gly110 peptide backbone to stabilize a flipped glycine conformation [50] when in the context of a fused benzopyridazine ring system (phthalazine). However, when the pyridazine ring is a core chemotype diversified with a pyridine, which has a single nitrogen atom with even greater basicity than pyridazine ring nitrogens, the pyridine ring nitrogen would be favored for protein H-bond interactions. This is what is observed in 4F9W and 4F9Y. Therefore, the pyridazine ring has the potential to be exploited as a biologically friendly fragment for kinase inhibitor development with either the potential to H-bond with the hinge region in a bidentate manner, thereby generating a glycine flip conformational change if the kinase is one of the few containing the appropriate sequence in the hinge region space, or the pyridazine ring can also be exploited as a molecular fragment placed in the context of a more basic substituent that forces the pyridazine ring to serve a different ligand role, such as a framework for diversifications that allows sampling of alternative space within kinases as observed in 4F9W and 4F9Y. The latter use of the pyridazine chemotype is not restricted to the localized sequence criteria so it is potentially more generalizable to other kinase targets. In either ligand role, the attractive biodistribution of pyridazine based molecular probes in complex biological systems is possible, depending on the molecular properties of the final ligand, as this aspect of pyridazines is a component of pharmacokinetics prior to target binding and engagement.

In conclusion, this report describes the development of selective chemical probes for *in vivo* studies of p38αMAPK and their use to demonstrate p38αMAPK is a key contributor to synaptic dysfunction and is amenable to therapeutic intervention. In addition, the insight gained from our co-crystallography studies under standardized conditions provides a foundation for future investigations of molecular recognition by small molecule probes and protein kinases.

## Supporting Information

Figure S1 Design strategy for structure-driven refinement of p38αMAPK inhibitor hit.
(DOC)

Figure S2 Kinetic analysis of MW01-11-108SRM inhibition of p38αMAPK.
(DOC)

Figure S3 Kinetic analysis of MW01-10-181SRM inhibition of p38αMAPK.
(DOC)

Scheme S1 Synthetic scheme for other p38αMAPK inhibitors. The synthetic scheme and experimental details for compounds in addition to MW108 and MW181 are given.
(DOC)

Table S1 MW01-11-108SRM kinome screen.
(DOC)

Table S2 MW01-10-181SRM kinome screen.
(DOC)
