# Supplementary material for: Development of Novel In Vivo Chemical Probes to Address CNS Protein Kinase Involvement in Synaptic Dysfunction
Source: PLoS One. 2013 Jun 26;8(6):e66226. doi: 10.1371/journal.pone.0066226 (PMC3694096; doi:10.1371/journal.pone.0066226)
Supplement: Figure S1 — Design strategy for structure-driven refinement of p38αMAPK inhibitor hit. (DOC) [file pone.0066226.s001.doc]

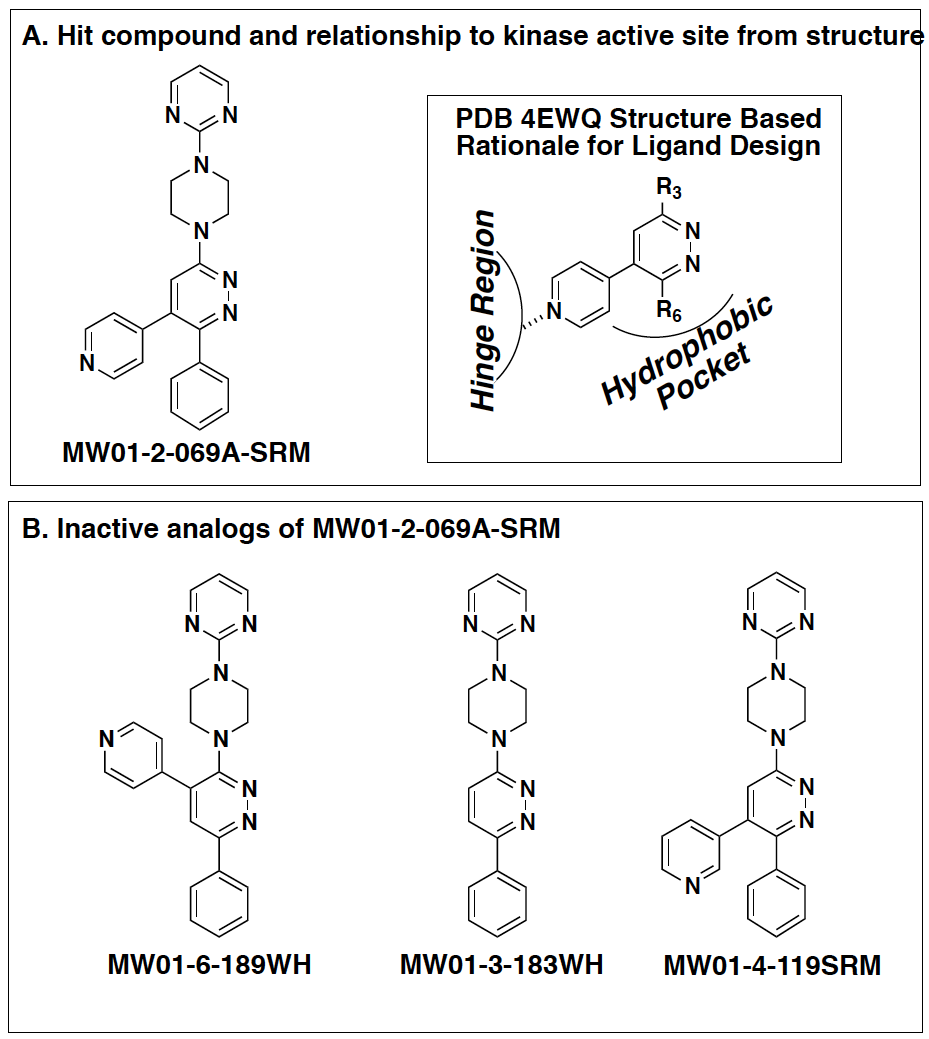


**Figure S1.** **Design strategy for structure-driven refinement of p38αMAPK inhibitor hit.** A) The synthesis and activity analysis campaign that delivered MW108 and MW181 started with a p38αMAPK inhibitor hit MW01-2-069A-SRM) that has *in vivo* CNS function [31]. The co-crystal structure of the hit in complex with human p38αMAPK (PDB 4EWQ) provided the structure-based rationale for ligand design. B) Initial synthesis and testing revealed a critical need for the pyridine substituent with its ring nitrogen at the correct position and the need for an aryl substituent at the vicinyl R6 position on the pyridazine ring. The ligand designs were filtered by pharmacoinformatics criteria based on prior success in CNS drug discovery, with a focus on key physical properties as a filter [6,7].
