# Supplementary material for: Development of Novel In Vivo Chemical Probes to Address CNS Protein Kinase Involvement in Synaptic Dysfunction
Source: PLoS One. 2013 Jun 26;8(6):e66226. doi: 10.1371/journal.pone.0066226 (PMC3694096; doi:10.1371/journal.pone.0066226)
Supplement: Figure S3 — Kinetic analysis of MW01-10-181SRM inhibition of p38αMAPK. (DOC) [file pone.0066226.s003.doc]

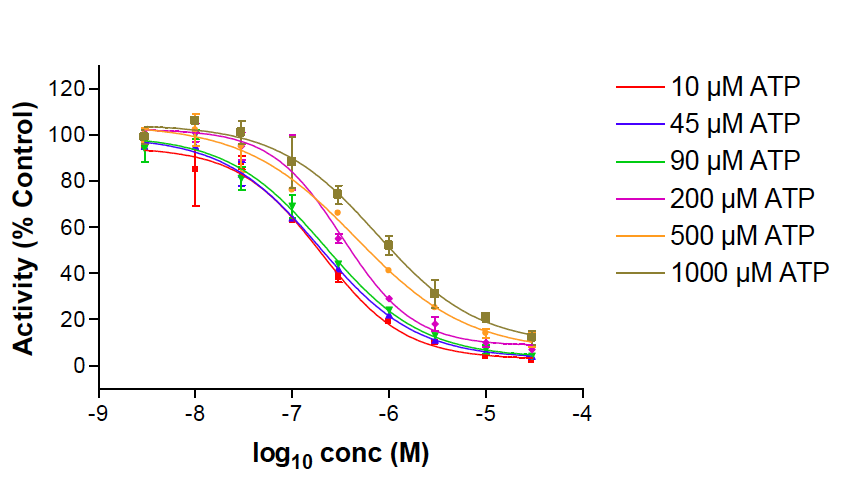

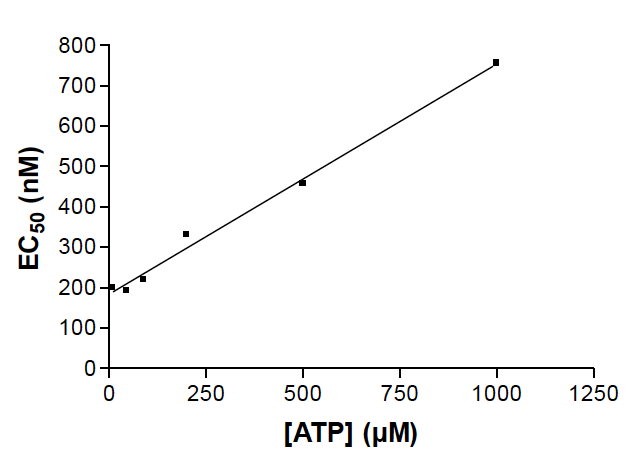

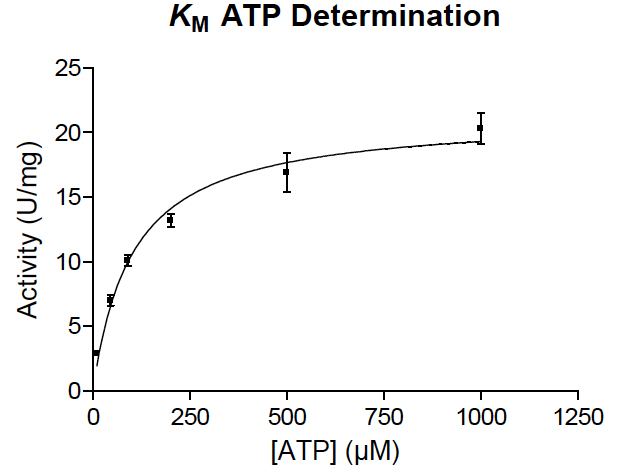


A.

B.

C.

**Figure S3. Kinetic analysis of MW01-10-181SRM inhibition of p38αMAPK.**

A) Kinase activity was measured at different ATP concentrations in the presence of increasing concentrations of MW181, and EC_50_ values determined at each ATP concentration. B) The EC_50_ values were re-plotted against the ATP concentration and the data fitted to a linear regression to obtain the y-axis intercept, which is taken as the *Ki*, which is 184 nM. C) Kinase activity determined in the absence of inhibitor allowed an estimation of the apparent Km for ATP of 102 μM.
