# Supplementary material for: Development of Novel In Vivo Chemical Probes to Address CNS Protein Kinase Involvement in Synaptic Dysfunction
Source: PLoS One. 2013 Jun 26;8(6):e66226. doi: 10.1371/journal.pone.0066226 (PMC3694096; doi:10.1371/journal.pone.0066226)
Supplement: Scheme S1 — Synthetic scheme for other p38αMAPK inhibitors. The synthetic scheme and experimental details for compounds in addition to MW108 and MW181 are given. (DOC) [file pone.0066226.s004.doc]

**Scheme S1. Synthetic scheme for other p38αMAPK inhibitors**

The synthetic scheme and experimental details for compounds described in this report in addition to MW108 and MW181 are given here.

All of the compounds described were made using previously described approaches (43), with a brief description below in sufficient detail to allow one skilled in the art to reproduce their production.

**1-Phenyl-2-(pyridin-4-yl)ethanone (1S):** Briefly, tetrahydrofuran (THF) in a round bottom flask was cooled (-78ºC), lithium diisopropylamide added to the flask with stirring under argon atmosphere, and a solution of 4-picoline (in THF) carefully added to the cooled flask under constant stirring. After one hour, a solution of *N*-methoxy-*N*-methylbenzamide (in THF) was added over a period of 30 min. Formation of the product was monitored by HPLC and TLC. The reaction mixture was warmed to ambient temperature, during which time the color changed from light yellow to orange, and the reaction quenched with crushed ice addition. THF was removed *in vacuo*, the reaction mixture was treated with saturated sodium bicarbonate solution, and repeat extraction with ethyl acetate was done in a separatory funnel. The combined organic extracts were treated with brine, dried over anhydrous magnesium sulfate, and concentrated under reduced pressure to yield an oily mixture. The crude mixture was purified by column chromatography on silica gel (200-400 mesh) with product elution using ethyl acetate: hexane (1:4 v/v). The product, ketone **1S**, was obtained as a bright yellow solid in 69% (gravimetric) yield, 90% purity by HPLC and a mass (ESI) of *m/z* (MeOH) = 198.18 (MH^+^).

**Ethyl 4-oxo-4-phenyl-3-(pyridin-4-yl)butanoate (2S):** Sodium hydride and chilled anhydrous THF (100 mL) were combined in a round bottom flask under argon at 0ºC. Compound **1S** in anhydrous THF was added drop wise over 1.5 h with constant stirring. After an additional 0.5 h, ethyl bromoacetate in anhydrous THF was added drop wise, the reaction mixture stirred at ambient temperature until completion as monitored by HPLC. The reaction mixture color changed from pale yellow to bright yellow. The reaction was quenched by addition of crushed ice, THF removed *in vacuo*, the reaction mix decanted into a saturated sodium bicarbonate solution, and the resulting solution subjected to repeat extraction with ethyl acetate using a separatory funnel. The combined organic extracts were treated with brine, dried over anhydrous sodium sulfate, and concentrated under reduced pressure to yield an oily residue. Product **2S** was obtained by silica gel (200-400 mesh) column chromatography using ethyl acetate: methanol (3:1 v/v) elution. Product **2S** was obtained as a beige solid in 74% (gravimetric) yield, 90% purity by HPLC, and a mass (ESI) of *m/z* (MeOH) = 284.75 (MH^+^).

**6-Phenyl-5-(pyridin-4-yl)-4,5-dihydropyridazin-3(2H)-one (3S):** Compound **2S** was mixed with ethanol (400 mL) in a round bottom flask, hydrazine hydrate was added, and the resulting solution was heated (90ºC) under reflux with continuous stirring until completion (~20h) as monitored by HPLC. The reaction mixture was cooled to ambient temperature and solvent removed under reduced pressure. The residue was treated with ethyl acetate followed by ether addition, and the solution taken to dryness by rotary evaporation. The white foam-like solid contained compound **3S**, with a mass (ESI) of *m/z* (MeOH) = 252.1(MH^+^), was used for the next step without further purification.

**6-Phenyl-5-(pyridin-4-yl)pyridazin-3-ol (4S):** Bromine in acetic acid was added drop wise under continuous stirring to a round bottom flask containing compound **3S**, the mixture refluxed at 70ºC until starting material was no longer detectable (~4h) as monitored by HPLC. The mixture was cooled to ambient temperature, poured over crushed ice, neutralized with a sodium bicarbonate solution, and subjected to repeat extraction with ethyl acetate. The combined organic layers were treated with brine, dried over sodium sulfate and evaporated under reduced pressure to give a deep brown solid. Compound **4S** was purified by silica gel (200-400 mesh) column chromatography using elution with ethyl acetate: methanol (9:1 v/v). Compound **4S** was obtained as a light brown solid in 68% (gravimetric) yield, 90% purity by HPLC, and a mass (ESI) *m/z* (MeOH) = 250.20 (MH^+^).

**6-Chloro-3-phenyl-4-(pyridin-4-yl)pyridazine (5S):** Compound **5S** was obtained following exactly our published protocol (43). Briefly, compound **4S** was suspended in acetonitrile, phosphorus oxychloride (Reagent Plus Grade, 99%) added, the mixture heated to 90ºC for 3h, cooled to ambient temperature, volatiles were removed *in vacuo*, the residual suspension poured into crushed ice, stirred for 3h at ambient temperature, and neutralized with 2.5N NaOH. The fine precipitate was subjected to repeat extraction with ethyl acetate, the combined organic phases subjected to drying over anhydrous magnesium sulfate and concentration *in vacuo*. Product **5S** was purified by silica gel (200-400 mesh) column chromatography using ethyl acetate: 1% methanol. Product **5S** was obtained as a white powder in 82% (gravimetric) yield, with an HPLC purity of 98% and a mass (ESI) *m/z* (MeOH) = 268.00 (MH^+^).

**Production of novel kinase inhibitors from common intermediate (5S):** The various novel kinase inhibitors were synthesized by reaction of a given amine with compound **5S** using the protocol described (43). Briefly, compound **5S** and 1-butanol were combined in a round bottom flask with the respective amine, heated to 110ºC for approximately 15 h, cooled to ambient temperature, treated with water, and the aqueous layer subjected to repeat extraction with dichloromethane. The combined organic layers were subjected to drying with anhydrous sodium sulfate and concentration *in vacuo*. The final products were purified by silica gel column chromatography using volatile solvents for elution and final processing.

**6-(4-methylpiperazin-1-yl)-3-phenyl-4-(pyridin-4-yl)pyridazine (6Sa = MW066):** Compound **5S** was reacted with 1-methylpiperazine and taken through the protocol above to give product **6Sa** as a beige powder in 89% (gravimetric) yield; MP: 190.2-190.7 ^o^C (uncorrected); 1H-NMR (CDCl3): δ 8.57 (dd, J = 1.55, 4.5 Hz, 2H); 7.34-7.24 (m, 5H); 7.12 (dd, J = 1.6, 4.2 Hz, 2H); 6.84 (s, 1H); 3.81 (t, J = 4.5, 4.6 Hz, 4H); 2.60 (s, 4H); 2.39 (s, 3H); HPLC (tr/purity): 9.3 min > 97% (HPLC method A); ESI m/z (MeOH): 332.1 (MH+); HRMS 331.1783 (calculated for C_20_H_22_N_5_ 331.1797).

**N, N-diethyl-6-phenyl-5-(pyridin-4-yl)pyridazin-3-amine (6Sb = MW177):** Compound **5S** was reacted with 10 equivalents of diethylamine and compound **6Sb** obtained by chromatographic elution with ethyl acetate: hexane (2:3 v/v) followed by crystallization in ethyl acetate and hexane to give beige crystals in 70% overall yield (gravimetric). MP: 121.5-122 ^o^C (uncorrected). ^1^H-NMR (CDCl_3_): δ 8.57 (d, J = 5.75 Hz, 2H); 7.34-7.23 (m, 5H); 7.14 (dd, J = 1.55, 4.45 Hz, 2H); 6.64 (s, 1H); 3.71 (dd, J= 7.1, 7.1 Hz, 4H); 1.29 (t, J= 7.1, 9 Hz, 6H); HPLC (tr/purity): 12.4 min, > 96% (HPLC method A); ESI m/z (MeOH): 305.10 (MH+); HRMS 304.1682 (calculated for C_19_H_20_N_4_ 304.1688).

**N-methyl-6-phenyl-N-propyl-5-(pyridin-4-yl)pyridazin-3-amine (6Sc = MW207):** Compound **5S** was reacted with N-methylpropane-1-amine, processed as above, and the final product obtained via chromatography using ethyl acetate: hexane (1:1 v/v) as solvent, followed by crystallization in ethyl acetate and hexane, to give the desired product **6Sc** as white crystals in 74% (gravimetric) overall yield. MP: 160.5-161 ^o^C (uncorrected). ^1^H-NMR (CD_3_OD): δ 8.47 (dd, J = 1.6, 4.55 Hz, 2H); 7.32-7.25 (m, 7H); 7.08 (s, 1H); 3.69 (t, J = 7.4, 7.4 Hz, 2H); 3.22 (s, 3H); 1.74 (m, 2H); 0.99 (t, J= 7, 7.45 Hz, 3H); HPLC (tr/purity): 12.8 min, > 96% (HPLC method A); ESI m/z (MeOH): 305.10 (MH+); HRMS 304.1699 (calculated for C_19_H_20_N_4_ 304.1688).

**N, N-dimethyl-6-phenyl-5-(pyridin-4-yl)pyridazin-3-amine (6Sd = MW105):**

Compound **5S** was reacted with six equivalents of 40% dimethylamine at 120ºC for 8 h and processed as above. The final product was obtained via crystallization from ethyl acetate and methanol to give the desired product **6Sd** as a light yellow crystalline solid in 90% yield (gravimetric). MP: 155.5-156 ^o^C (uncorrected). ^1^H-NMR (CDCl_3_): δ 8.57 (d, J = 5.8 Hz, 2H); 7.34-7.24 (m, 5H); 7.14 (dd, J = 1.5, 4.65, 2H); 6.72 (s, 1H); 3.27 (s, 6H); HPLC (tr/purity): 10.3 min > 98% (HPLC method A); ESI m/z (MeOH): 277.14 (MH+); HRMS 276.1384 (calculated for C_17_H_16_N_4_ 276.1375).

**Production of hydrochloride hydrate forms of 6Sa, 6Sb, 6Sc and 6Sd:** This was done following the protocol of Hu et al. (43). Under these conditions for the studies described here, elemental analyses of each product indicated a mole ratio of HCl:compound of ~2. **MW066 hydrochloride hydrate:** EA calculated for C_20_H_23_Cl_2_N_5_: C, 59.41; H, 5.73; Cl, 17.54; N, 17.32; experimentally found C, 59.08; H, 5.69; Cl, 17.14; N, 17.11; O, 1.03. **MW-177 hydrochloride hydrate:** EA calculated for C_19_ H_26_ Cl_2_ N_4_ O_2_: C, 55.21; H, 6.34; Cl, 17.15; N, 13.55; O, 7.74; experimentally found C, 56.30; H, 5.85; Cl, 20.23; N, 13.77; O, 3.32. **MW207 hydrochloride hydrate:** EA calculated for C_19_ H_26_ Cl_2_ N_4_ O_2_: C, 55.21; H, 6.34; Cl, 17.15; N, 13.55; O, 7.74; experimentally found C, 55.49; H, 6.17; Cl, 17.92; N, 13.60; O, 3.58. **MW105 hydrochloride hydrate:** EA calculated for C_17_H_24_Cl_2_N_4_O_3_: C, 50.63; H, 6.00; Cl, 17.58; N, 13.89; O, 11.90; experimentally found: C, 50.75; H, 5.89; Cl, 17.75; N, 13.90; O, 12.20.
