# Supplementary material for: Development of Novel In Vivo Chemical Probes to Address CNS Protein Kinase Involvement in Synaptic Dysfunction
Source: PLoS One. 2013 Jun 26;8(6):e66226. doi: 10.1371/journal.pone.0066226 (PMC3694096; doi:10.1371/journal.pone.0066226)
Supplement: Table S1 — MW01-11-108SRM kinome screen. (DOC) [file pone.0066226.s005.doc]

**Table S1. MW01-11-108SRM Kinome Screen**

**★**Initial profile screen done at 20,000 nM of inhibitor as described in Materials and Methods

****IC_50_ determined for target hits (<40% activity remaining) as described in Materials and Methods

Estimated *Ki* (Table 1 main text) determined for confirmed positives (IC_50_ <1,000 nM)

| **Kinase Name** | **Accession Number** | **% Activity★** | **IC_50_ (nM) ** |
| --- | --- | --- | --- |
| Abl | U07563 | 99 |  |
| Abl(m) | J02995 | 89 |  |
| Abl (H396P) | U07563 | 94 |  |
| Abl (M351T) | U07563 | 106 |  |
| Abl (Q252H) | U07563 | 105 |  |
| Abl(T315I) | U07563 | 91 |  |
| Abl(Y253F) | U07563 | 98 |  |
| ACK1 | NM_005781 | 100 |  |
| ALK | U62540 | 105 |  |
| ALK4 | NM_004302 | 95 |  |
| Arg | NM_005158 | 95 |  |
| AMPKα1 | AF100763 (a1)/NM_006253 (b1)/NM_002733 (g1) | 101 |  |
| AMPKα2 | NM_006252(a2)/NM_006253 (b1)/NM_002733 (g1) | 102 |  |
| Arg(m) | XM_136360 | 86 |  |
| ARK5 | NM_014840 | 98 |  |
| ASK1 | NM_005923 | 103 |  |
| Aurora-A | NM_003600 | 118 |  |
| Aurora-B | NM_004217 | 134 |  |
| Aurora-C | AB017332 | 113 |  |
| Axl | NM_021913 | 106 |  |
| Blk | NM_001715 | 88 |  |
| Blk(m) | M30903 | 88 |  |
| Bmx | NM_001721 | 87 |  |
| BRK | NM_005975 | 85 |  |
| BrSK1 | NM_032430 | 94 |  |
| BrSK2 | AF533878 | 88 |  |
| BTK | NM_000061 | 97 |  |
| BTK(R28H) | NM_000061 | 101 |  |
| CaMKI | NM_003656 | 101 |  |
| CaMKIIβ | AF081572 | 96 |  |
| CaMKIIγ | NM_172171 | 99 |  |
| CaMKIδ | NM_153498 | 97 |  |
| CaMKIIδ | NM_172115 | 100 |  |
| CaMKIV | D30742 | 100 |  |
| CDK1/cyclinB | NM_001786(CDK1)/P14635 (cyclinB) | 90 |  |
| CDK2/cyclinA | M68520(CDK2)/X51688(cyclinA) | 79 |  |
| CDK2/cyclinE | M68520(CDK2)/NM_001238(cyclinE) | 108 |  |
| CDK3/cyclinE | X66357(CDK3)/NM_001238(cyclinE) | 91 |  |
| CDK5/p25 | X66364(CDK5)/X80343(p25) | 106 |  |
| CDK5/p35 | X66364(CDK5)/X80343(p35) | 99 |  |
| CDK6/cyclinD3 | X66365(CDK6)/M90814(cyclinD3) | 85 |  |
| CDK7/cyclinH/MAT1 | X79193(CDK7)/U11791(cyclinH)/X87843(MAT1) | 98 |  |
| CDK9/cyclin T1 | AF517840(CDK9)/NM_001240(cyclinT1) | 95 |  |
| CHK1 | O14757 | 94 |  |
| CHK2 | NP_009125 | 97 |  |
| CHK2(I157T) | NP_009125 | 101 |  |
| CHK2(R145W) | NP_009125 | 97 |  |
| CK1γ1 | NM_022048 | 101 |  |
| CK1γ2 | NM_001319 | 91 |  |
| CK1γ3 | NM_004384 | 100 |  |
| CK1δ | NM_001893 | 33 | 6118 |
| CK2 | J02853 (alpha)/NM_001320 (beta) | 106 |  |
| CK2α2 | NM_001896 | 114 |  |
| CLK2 | NM_003993 | 103 |  |
| CLK3 | NM_003992 | 102 |  |
| cKit | X06182 | 104 |  |
| cKit(D816V) | X06182 | 103 |  |
| cKit(D816H) | X06182 | 109 |  |
| cKit(V560G) | X06182 | 95 |  |
| cKit(V654A) | X06182 | 106 |  |
| CSK | X60114 | 93 |  |
| c-RAF | P04049 | 27 | 9116 |
| cSRC | P12931 | 125 |  |
| DAPK1 | NM_004938 | 92 |  |
| DAPK2 | NM_014326 | 101 |  |
| DCAMKL2 | NM_152619 | 109 |  |
| DDR2 | NM_006182 | 11 | 2865 |
| DMPK | NM_004409 | 108 |  |
| DRAK1 | NM_004760 | 86 |  |
| DYRK2 | NM_003583 | 108 |  |
| eEF-2K | NM_013302 | 108 |  |
| EGFR | X00588 | 79 |  |
| EGFR(L858R) | X00588 | 48 |  |
| EGFR(L861Q) | X00588 | 49 |  |
| EGFR(T790M) | X00588 | 68 |  |
| EGFR(T790M,L858R) | X00588 | 40 |  |
| EphA1 | AH007960 | 97 |  |
| EphA2 | NM_004431 | 83 |  |
| EphA3 | NM_005233 | 101 |  |
| EphA4 | NM_004438 | 97 |  |
| EphA5 | NM_004439 | 100 |  |
| EphA7 | NM_004440 | 35 | 18894 |
| EphA8 | NM_020526 | 95 |  |
| EphB2 | D31661 | 36 | 16193 |
| EphB1 | NM_004441 | 92 |  |
| EphB3 | NM_004443 | 96 |  |
| EphB4 | AY056047 | 54 |  |
| ErbB4 | NM_005235 | 52 |  |
| FAK | NM_005607 | 95 |  |
| Fer | NM_005246 | 97 |  |
| Fes | X06292 | 111 |  |
| FGFR1 | NM_0023110 | 93 |  |
| FGFR1(V561M) | NM_0023110 | 90 |  |
| FGFR2 | NM_000141 | 101 |  |
| FGFR2(N549H) | NM_000141 | 76 |  |
| FGFR3 | M58051 | 86 |  |
| FGFR4 | L03840 | 89 |  |
| Fgr | M19722 | 92 |  |
| Flt1 | AF063657 | 38 | >30,000 |
| Flt3(D835Y) | NM_004119 | 90 |  |
| Flt3 | NM_004119 | 108 |  |
| Flt4 | NM_182925 | 11 | 4914 |
| Fms | U63963 | 98 |  |
| Fms(Y969C) | U63963 | 100 |  |
| Fyn | P06241 | 95 |  |
| GCK | BC047865 | 101 |  |
| GRK5 | NM_005308 | 110 |  |
| GRK6 | NM_001004106 | 98 |  |
| GRK7 | NM_139209 | 98 |  |
| GSK3α | NM_019884 | 90 |  |
| GSK3β | P49841 | 97 |  |
| Haspin | NM_031965 | 87 |  |
| Hck | NM_002110 | 93 |  |
| Hck activated | NM_002110 | 97 |  |
| HIPK1 | NM_152696 | 98 |  |
| HIPK2 | AF326592 | 107 |  |
| HIPK3 | NM_005734 | 104 |  |
| IGF-1R | X04434 | 137 |  |
| IGF-1R, activated | X04434 | 104 |  |
| IKKα | AF012890 | 105 |  |
| IKKβ | AF080158 | 98 |  |
| IR | NM_000208 | 100 |  |
| IR, activated | NM_000208 | 103 |  |
| IRR | NM_014215 | 94 |  |
| IRAK1 | NM_001569 | 97 |  |
| IRAK4 | AF445802 | 91 |  |
| Itk | NM_005546 | 90 |  |
| JAK2 | NM_004972 | 98 |  |
| JAK3 | AF513860 | 90 |  |
| JNK1α1 | L26318 | 96 |  |
| JNK2α2 | P45984 | 76 |  |
| JNK3 | NM_138980 | 5 | 1257 |
| KDR | NM_002253 | 87 |  |
| Lck | P06239 | 62 |  |
| Lck activated | P06239 | 70 |  |
| LIMK1 | NM_002314 | 106 |  |
| LKB1 | NM_000455 | 102 |  |
| LOK | NM_005990 | 48 | >30,000 |
| Lyn | M16038 | 100 |  |
| Lyn(m) | M64608 | 97 |  |
| MAPK1 | NM_002746 | 82 |  |
| MAPK2 | NM_002745 | 113 |  |
| MAPK2(m) | P27703 | 109 |  |
| MAPKAP-K2 | P49137 | 103 |  |
| MAPKAP-K3 | NM_004635 | 102 |  |
| MEK1 | Q02750 | 130 |  |
| MARK1 | AF154845 | 113 |  |
| MELK | NM_014791 | 90 |  |
| Mer | NM_006343 | 98 |  |
| Met | J02958 | 118 |  |
| Met(D1246H) | J02958 | 94 |  |
| Met(D1246N) | J02958 | 80 |  |
| Met(M1268T) | J02958 | 91 |  |
| Met(Y1248C) | J02958 | 84 |  |
| Met(Y1248D) | J02958 | 93 |  |
| Met(Y1248H) | J02958 | 88 |  |
| MINK | NM_015716 | 48 |  |
| MKK4(m) | P47809 | 108 |  |
| MKK7β | O14733 | 122 |  |
| MLCK | NM_053025 | 94 |  |
| MLK1 | NM_033141 | 98 |  |
| Mnk2 | NM_017572 | 99 |  |
| MRCKα | NM_003607 | 107 |  |
| MRCKβ | NM_006035 | 99 |  |
| MSK1 | AF074393 | 97 |  |
| MSK2 | AJ010119 | 91 |  |
| MSSK1 | NM_014370 | 86 |  |
| MST1 | NM_006282 | 105 |  |
| MST2 | U60206 | 98 |  |
| MST3 | BC035578 | 98 |  |
| mTOR | NM_004958 | 92 |  |
| mTOR/FKBP12 | NM_004958(mTOR)/NM_054014(FKBP12) | 100 |  |
| MuSK | NM_005592 | 104 |  |
| NEK2 | NM_002497 | 96 |  |
| NEK3 | NM_002498 | 98 |  |
| NEK6 | NM_014397 | 103 |  |
| NEK7 | AB062450 | 108 |  |
| NEK11 | BC028587 | 108 |  |
| NLK | NM_016231 | 20 | 1215 |
| p70S6K | M60724 | 92 |  |
| PAK2 | U24153 | 103 |  |
| PAK4 | NM_005884 | 104 |  |
| PAK3 | AB102659 | 78 |  |
| PAK5 | NM_020341 | 102 |  |
| PAK6 | NM_020168 | 99 |  |
| PAR-1Bα | AF387638 | 97 |  |
| PASK | NM_015148 | 88 |  |
| PDGFRα | M21574 | 106 |  |
| PDGFRα(D842V) | M21574 | 89 |  |
| PDGFRα(V561D) | M21574 | 114 |  |
| PDGFRβ | J03278 | 99 |  |
| PDK1 | O15530 | 104 |  |
| PhKγ2 | NM_000294 | 98 |  |
| PI3 Kinase (p110β/p85α) | NM_006219 | 100 |  |
| PI3 Kinase (p120γ) | NM_002649 | 101 |  |
| PI3 Kinase (p110δ/p85α) | NM_005026 | 100 |  |
| PI3 Kinase (p110α/p85α)(m) | NM_006218 | 101 |  |
| PI3 Kinase (p110α/p65α)(m) | NM_006218 | 101 |  |
| PI3 Kinase (p110α (E545K)/p85α)(m) | NM_006218 | 99 |  |
| PI3 Kinase (p110δ/p85α)(m) | NM_008840 | 100 |  |
| PI3 Kinase (p110α (E542K)/p85α) | NM_006218 | 99 |  |
| PI3 Kinase (p110α (H1047R)/p85α | NM_006218 | 99 |  |
| PI3 Kinase (p110 β/p85β)(m) | NM_006219 | 101 |  |
| PI3 Kinase (p110α (E542K)/p85α) | NM_006218 | 99 |  |
| PI3 Kinase (p110α (H1047R)/p85α) | U79143 | 99 |  |
| PI3 Kinase (p110α (E545K)/p85α) | NM_006218 | 98 |  |
| PI3 Kinase (p110 β/p85α)(m) | NM_029094 | 99 |  |
| PI3 Kinase (p110α/p85α) | NM_006218 | 98 |  |
| PI3 kinase (p110α/p65α) | NM_006218 | 99 |  |
| PI3K-C2α | NM_002645 | 29 | >30,000 |
| PI3K-C2γ | BC130277 | 50 |  |
| PIP4K2α | NM_005028 | 96 |  |
| PIP5K1α | U78577 | 104 |  |
| PIP5K1γ | NM_012398 | 104 |  |
| Pim-1 | M27903 | 103 |  |
| Pim-2 | U77735 | 93 |  |
| Pim-3 | AB114795 | 105 |  |
| PKA | X07767 | 74 |  |
| PKBα | P31749 | 99 |  |
| PKBβ | M95936 | 98 |  |
| PKBγ | AF124141 | 92 |  |
| PKCα | X52479 | 92 |  |
| PKCβI | X06318 | 92 |  |
| PKCβII | X07109 | 87 |  |
| PKCγ | XM_017991 | 93 |  |
| PKCδ | L07861 | 101 |  |
| PKCε | X65293 | 90 |  |
| PKCη | NM_006255 | 88 |  |
| PKCι | NM_002740 | 92 |  |
| PKCμ | NM_002742 | 70 |  |
| PKCθ | L07032 | 107 |  |
| PKCζ | BC014270 | 103 |  |
| PKD2 | NM_016457 | 78 |  |
| PKG1α | D45864 | 93 |  |
| PKG1β | NM_006258 | 96 |  |
| Plk1 | NM_005030 | 96 |  |
| Plk3 | NM_004073 | 103 |  |
| PRAK | O60491 | 94 |  |
| PRK2 | NM_006256 | 103 |  |
| PrKX | NM_005044 | 109 |  |
| PTK5 | NM_002031 | 80 |  |
| Pyk2 | S80542 | 106 |  |
| Ret | NM_000323 | 94 |  |
| Ret (V804L) | NM_000323 | 102 |  |
| Ret(V804M) | NM_000323 | 85 |  |
| RIPK2 | NM_003821 | 57 |  |
| ROCK-I | NM_005406 | 89 |  |
| ROCK-II | NM_004850 | 99 |  |
| ROCK-II(r) | NM_013022 | 97 |  |
| Ron | NM_002447 | 91 |  |
| Ros | M34353 | 87 |  |
| Rse | D17517 | 117 |  |
| Rsk1 | NM_002953 | 93 |  |
| Rsk1(r) | M99169 | 91 |  |
| Rsk2 | NM_004586 | 92 |  |
| Rsk3 | XM_004469 | 83 |  |
| Rsk4 | NM_014496 | 96 |  |
| *SAPK2a | NM_139012 | 2 | 236 |
| *SAPK2a (T106M) | NM_139012 | 59 |  |
| *SAPK2b | NM_002751 | 18 | 3047 |
| *SAPK3 | P53778 | 87 |  |
| *SAPK4 | O15264 | 93 |  |
| SGK | AF153609 | 88 |  |
| SGK2 | NM_016276 | 92 |  |
| SGK3 | NM_013257 | 104 |  |
| SIK | NM_173354 | 91 |  |
| Snk | NM_006622 | 98 |  |
| Src(1-530) | K03218 | 101 |  |
| Src(T341M) | K03218 | 92 |  |
| SRPK1 | NM_003137 | 114 |  |
| SRPK2 | NM_182691 | 95 |  |
| STK33 | NM_030906 | 106 |  |
| Syk | L28824 | 88 |  |
| TAK1 | NM_003188 | 102 |  |
| TAO1 | NM_020791 | 92 |  |
| TAO2 | NM_016151 | 81 |  |
| TAO3 | NM_016281 | 73 |  |
| TBK1 | NM_013254 | 96 |  |
| Tec(h) activated | NM_003215 | 89 |  |
| TGFBR1 | NM_004612 | 76 |  |
| Tie2 | NM_000459 | 81 |  |
| Tie2(R849W) | NM_000459 | 77 |  |
| Tie2(Y897S) | NM_000459 | 95 |  |
| TLK2 | BC044925 | 99 |  |
| TrkA | NM_002529 | 91 |  |
| TrkB | U12140 | 78 |  |
| TSSK1 | NM_032028 | 101 |  |
| TSSK2 | NM_053006 | 101 |  |
| Txk | NM_003328 | 98 |  |
| ULK2 | NM_014683 | 100 |  |
| ULK3 | NM_015518) | 95 |  |
| WNK2 | NM_006648 | 101 |  |
| WNK3 | NM_020922 | 91 |  |
| VRK2 | NM_006296 | 83 |  |
| Yes | M15990 | 63 |  |
| ZAP-70 | P43403 | 92 |  |
| ZIPK | AB007144 | 93 |  |
| *SAPK=p38MAPK |  |  |  |
